# Spatial distribution, contamination levels, and health risk of potentially toxic elements in surface soils of an industrial—urban area in northwestern Mexico: a case study from La Paz, Baja California Sur

**DOI:** 10.1007/s10653-025-02918-7

**Published:** 2025-12-09

**Authors:** Benedetto Schiavo, Jaqueline Valenzuela-Meza, Daisy Valera-Fernández, Claudio Inguaggiato, Diana Meza-Figueroa, Ofelia Morton-Bermea

**Affiliations:** 1 Instituto de Geofísica, Universidad Nacional Autónoma de México, Mexico City, Mexico; 2 Centro de Energía Renovable y Calidad Ambiental, La Paz, Baja California Sur, Mexico; 3 Instituto de Geología, Universidad Nacional Autónoma de México, Mexico City, Mexico; 4 Departamento de Geología, Centro de Investigación Científica y de Educación Superior de Ensenada, Baja California (CICESE), Ensenada, Mexico; 5 Departamento de Geología, Universidad de Sonora, Hermosillo, Mexico

**Keywords:** Trace elements, Pollution assessment, Geochemical characterization, Magnetic susceptibility, Source identification, Ecological risk

## Abstract

This study presents a comprehensive evaluation of potentially toxic elements (PTEs) contamination in surface soils of La Paz, Baja California Sur, Mexico, integrating geochemical, mineralogical, and magnetic analyses to identify contamination levels, spatial patterns, and potential health risks. Concentrations of Zn, V, Cr, Pb, Cu, Ni, As, Co, Sb, and Cd frequently exceeded local geochemical background values, with V and Ni particularly elevated near the heavy fuel oil fired (CTPP) and heavy fuel oil/diesel (CCI) power plants, consistent with emissions from fuel combustion. Spatial distribution analysis revealed that point sources dominated the signatures of V and Ni, while diffuse sources, such as vehicular traffic, contributed to elevated Sb, Zn, and Cu, especially along highways and in residential parks. Mixed-source elements (Cr, Pb, Co, As) showed overlapping industrial and urban signatures, with prevailing north-northwest winds likely enhancing contaminant dispersion. Soil color attributes and low frequency—dependent susceptibility indicated the predominance of coarse anthropogenic magnetic particles. Human health risk assessment showed no non-carcinogenic risk for adults, but hazard indices for children exceeded the safe threshold across all land uses, with the highest value at CTPP (HI = 2.22). Total cancer risk values remained within acceptable limits, although As contributions near CTPP approached the upper boundary. These findings highlight the persistent influence of industrial and urban activities on soil quality and underscore the importance of targeted monitoring and mitigation strategies to protect at-risk populations.

**Supplementary Information:**

The online version contains supplementary material available at 10.1007/s10653-025-02918-7.

## Introduction

Soil, as a dynamic and fundamental component of terrestrial ecosystems, plays a critical role in regulating environmental quality and safeguarding both human and ecological health. However, increasing anthropogenic pollution, particularly from industrial activities such as mining, smelting, manufacturing, and fossil fuel combustion, has led to the accumulation of potentially toxic elements (PTEs) in urban and peri-urban soils. Among these, heavy metals and metalloids such as arsenic (As), cadmium (Cd), chromium (Cr), copper (Cu), lead (Pb), nickel (Ni), and zinc (Zn) are of major concern due to their persistence, non-biodegradability, and potential to cause adverse effects even at low concentrations (Reis & Dindaroğlu, 2024). Likewise, Schiavo et al. (2023) highlighted that metals in urban dust and soil can exert significant oxidative potential, representing a critical environmental stressor with implications for chronic diseases. These toxicological effects are largely driven by transition metals such as Cu, Cr, and Ni, which may derive from both natural geogenic inputs (e.g., volcanic emission and soil erosion)(Schiavo et al., 2024) and anthropogenic sources (e.g., industrial emissions and traffic-related activities)(Anwar et al., 2025). Once mobilized in soils, these elements can trigger oxidative stress in human cells and tissues, contributing to cellular damage and increasing the risk of chronic diseases (Haidar et al., 2023; Jomova & Valko, 2011).

Industrial operations are recognized as major contributors to metal pollution in soil surface horizons (Obiri-Nyarko et al., 2024; Parlak et al., 2023). Processes such as ore refining, fossil fuel burning, waste disposal, and emissions from energy plants release significant quantities of PTEs into the environment (Botsou et al., 2022; Kravchenko et al., 2025), which are then deposited onto surrounding soils via atmospheric fallout, runoff, or direct discharge. These contaminants can persist in the topsoil for extended periods, thereby posing long-term ecological risks and potential health hazards through multiple exposure pathways, including ingestion, inhalation of resuspended particles, and dermal contact (Botsou et al., 2022). The accumulation of heavy metals in soil can adversely affect microbial communities, alter physicochemical properties, reduce fertility, and compromise the productivity of urban green spaces (Edwards, 2002). From a human health perspective, the presence of toxic metals in residential or recreational areas is particularly alarming. Children are especially vulnerable due to their behavioral patterns and physiological characteristics, making risk assessment a necessary component of environmental monitoring programs (Tepanosyan et al., 2017).

Numerous investigations across Asia, Europe, Africa, and Latin America have consistently reported elevated concentrations of PTEs in urban soils located near metallurgical plants, power stations, industrial landfills, and major roadways (Yang et al., 2016; Aminiyan et al., 2021; Botsou et al., 2022; Ahamad et al., 2021). These environments are frequently subjected to chronic emissions from industrial processes, fossil fuel combustion, and the atmospheric deposition of particulates, leading to persistent PTE accumulation in soils. In rapidly industrializing regions of China, Yang et al. (2016) documented excessive levels of As, Cr, and Pb, primarily linked to both historical and ongoing smelting activities, with anthropogenic inputs markedly surpassing natural background levels. Similarly, Aminiyan et al. (2021) found severe Cd, Pb, and Zn enrichment in soils surrounding a copper smelter in Iran, where atmospheric deposition and inadequate waste management facilitated metal dispersion into nearby agricultural and residential areas. In industrialized urban zones, the mobility of certain metals such as Pb and Zn has been shown to be high, as observed by Botsou et al. (2022) in Greece, raising concerns over their remobilization potential and associated risks. Studies in South Asia have revealed comparable contamination patterns: Ahamad et al. (2021) reported that soils adjacent to industrial landfills in Pakistan exhibited notable ecological risks, particularly from Hg and Cd, with potential health implications for exposed populations. Collectively, these studies highlight the role of industrial emissions, waste disposal practices, and transportation-related activities as dominant drivers of soil metal contamination in urban and peri-urban settings, underscoring the importance of integrating geochemical data with risk assessment frameworks to suggest and design mitigation strategies.

In many urban-industrial settings, particularly in low- and middle-income countries, limited regulatory enforcement and insufficient environmental monitoring exacerbate the accumulation of heavy metals in soils (Caravanos et al., 2014). Mexico, like other rapidly developing nations, faces mounting environmental pressures due to urbanization, population growth, vehicular emissions, and the expansion of energy infrastructure. Although several studies have assessed heavy metal contamination in soils across Mexico (Morton-Bermea et al., 2009; Pérez-Vázquez et al., 2015; Ponce-Hernández et al., 2025), there remains a significant knowledge gap in the Baja California Peninsula, particularly concerning the distribution, sources, and risks of metal(loid)s in urban and peri-urban soils influenced by energy infrastructure. In addition, most prior investigations have focused solely on concentration levels without integrating spatial analysis or health risk modeling. An interdisciplinary approach that incorporates geochemical analysis, contamination indices, risk assessment, and geospatial tools is crucial for understanding the extent and implications of pollution (He et al., 2021), and for developing evidence-based strategies for land use planning and environmental management (Omo-Okoro et al., 2025).

To address these gaps, this study aims to assess the concentrations, spatial patterns, and potential ecological and human health risks of PTEs in surface soils from urban areas, energy plant surroundings, and nearby recreational zones in La Paz. Ten PTEs (Zn, V, Cr, Pb, Cu, Ni, As, Co, Sb, Cd) were selected according to their environmental and toxicological relevance, their occurrence in urban and industrial soils, and their ability to discriminate between potential emission sources (Li et al., 2020). This study employs high-precision analytical techniques and contamination indices to assess the total concentrations and contamination levels of PTEs in surface soils. Health risk was evaluated using standard international models including both non-carcinogenic and carcinogenic pathways for adults and children. Furthermore, geostatistical analysis was employed to explore spatial distribution patterns of PTEs and to identify contamination hotspots associated with anthropogenic sources such as traffic corridors and industrial emissions. By integrating analytical chemistry, environmental modeling, and spatial analysis, the findings from this study contribute valuable data for local environmental authorities and offer a scientific basis for future soil remediation strategies and pollution prevention programs.

## Materials and methods

### Study area

**Fig. 1:**
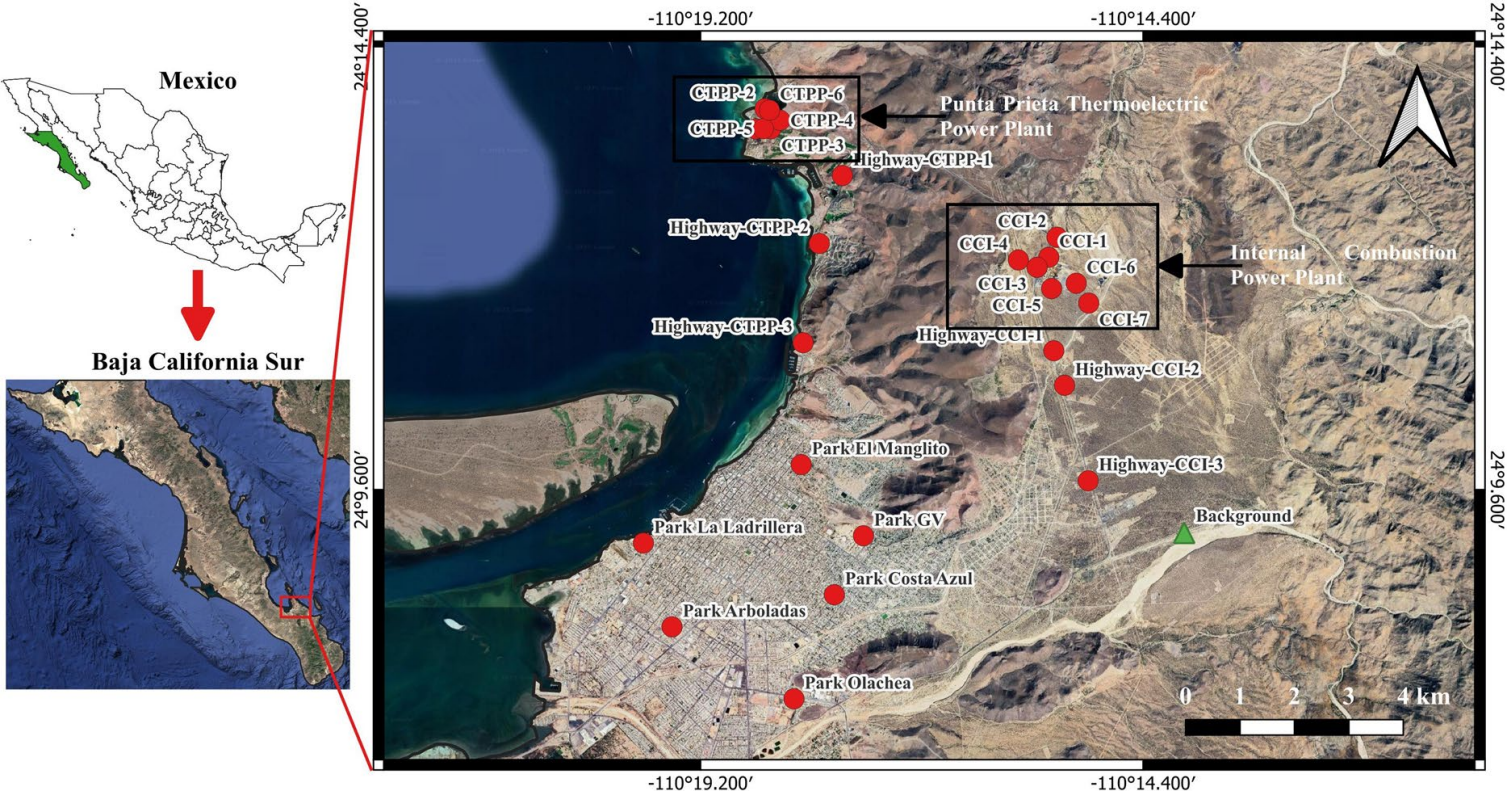
Location of the 26 soil sampling sites in La Paz, Baja California Sur, Mexico, including areas near the Punta Prieta Thermoelectric Power Plant (CTPP), the internal combustion power plant (CCI), major highways, and urban parks. The background site (green triangle) is indicated for baseline reference. The orange rectangle highlights the urban area of La Paz city

La Paz is the capital city of the state of Baja California Sur (Fig. 1), located in northwestern Mexico along the Gulf of California (24°08′N, 110°18′W). The urban area spans approximately 20,275 km^2^ with a population of around 250,000 inhabitants in the city proper (292,241 in the municipality) as of 2020, representing a 16% increase since 2010 (INEGI, 2015). The climate is arid and semi-hot, with mean annual temperatures ranging from 23 to 25 °C, and summer temperatures frequently exceeding 40 °C during the hottest months (June to August). Annual precipitation is low, averaging around 180 mm, mostly concentrated between August and September (INAFED, 2015). The area is subject to seasonal winds and dust resuspension, contributing to the dispersion of airborne pollutants.

The study area includes urban zones within La Paz and its surroundings, with particular focus on sites influenced by major roads, industrial activity, and two heavy fuel oil power plants: Punta Prieta Thermoelectric Power Plant (CTPP) and the Internal Combustion Power Plant (CCI). The location of major industrial facilities (CTPP and CCI) relative to the sampling points is shown in Fig. S1.The city has experienced significant urban expansion and vehicular growth over the last decades, raising concerns over environmental contamination, especially related to heavy metals in soils and road dust. Between 1950 and 2020, the city experienced a sustained annual population growth rate of about 4.5%, reflecting accelerated urban expansion (INEGI, 2020; Ivanova et al., 2015). This rapid urbanization has been accompanied by a notable increase in vehicle use. In 2020, 50.7% of the population in Baja California Sur reported using a private vehicle (car, truck, or motorcycle) as their main mode of transport to work, indicating significant motorization across the region (INEGI, 2020).

Geologically, the region belongs to the Peninsular Ranges province and is composed mainly of volcanic and intrusive igneous rocks, which can naturally influence background metal concentrations (Drake et al., 2017). The predominant soil types in the area are Regosols and Leptosols, characterized by weak profile development, coarse textures, and low organic matter content (Bocco et al., 2001; INEGI, 2001). However, anthropogenic activities are expected to be the primary source of metal enrichment in the surface soils, particularly near roadways, industrial corridors, and energy generation facilities.

### Sampling and analytical procedure

Soil sampling (n = 26) was conducted between 17 and 26 August 2022 in the urban area of La Paz, Baja California Sur, and its surroundings. The sampling design considered four groups of sites: (i) areas surrounding the CCI and along the highway–CCI corridor, (ii) areas around the CTPP and along the highway–CTPP corridor, (iii) urban parks within the city, and (iv) background site located outside the urban zone (Fig. 1). Sampling locations were selected following a predefined sampling plan and were conditioned by site accessibility. For the power plant sites, samples were collected as close as possible to the facility perimeter at cardinal points, with additional samples taken approximately 500 m away in the same direction. For the road corridors (highway–CCI and highway–CTPP), sampling was carried out at different points along each route, maintaining a minimum distance of 15 m from the road edge. In the case of urban parks, samples were collected near the central areas of the parks, in zones surrounded by residential and commercial areas. A background soil sample was collected in an area located outside the urban footprint, at sufficient distance from traffic corridors, industrial facilities, and other potential anthropogenic emission sources, to represent the natural geochemical baseline of the region. At each location, surface soil (0–5 cm depth) was collected after removing stones and coarse debris. Heavy metals tend to accumulate in the surface soil, showing limited mobility toward deeper horizons (Kabata-Pendias, 2000; Zhang et al., 2011). This upper horizon reflects recent atmospheric deposition and represents the layer with the greatest potential for direct human exposure (Wei & Yang, 2010). Approximately 300 g of soil per sample was collected using a clean plastic scoop and stored in labeled polyethylene bags. Each sample was georeferenced and photographs of the sampling sites were taken to document conditions.

The concentrations of PTEs (As, Cd, Co, Cr, Cu, Ni, Pb, Sb, V, and Zn) in surface soil samples (< 200 µm fraction) were determined using inductively coupled plasma–mass spectrometry (ICP–MS, iCAP Qc, Thermo Fisher Scientific, USA) at the Institute of Geophysics, National Autonomous University of Mexico (IGF-UNAM). Approximately 0.5 g of homogenized soil was digested following a total acid digestion protocol with a mixture of concentrated HNO₃, HCl, and HF (9:3:2 mL) in Teflon vessels (Schiavo et al., 2024). The digestion was carried out in a microwave-assisted system (ETHOS ONE, Milestone) equipped with a PRO-24 rotor to ensure complete dissolution of silicate and oxide phases. A total digestion approach was selected because the objective of this study was to quantify the overall concentration and total elemental burden of PTEs in soils. Total digestion allows direct comparison with soil-quality guidelines, which are typically based on total metal concentrations, and provides a robust basis for identifying source-related enrichment patterns. After cooling, the digests were evaporated to near dryness and dissolved in 50 mL of 3% ultrapure HNO_3_ prior to ICP–MS analysis. Instrumental calibration was performed using a 14-point multi-element standard curve (0–500 μg L^−1^; ICP-MS-68A, High-Purity Standards). Accuracy was assessed by the analysis of the standard reference material NIST SRM 1648a (urban particulate matter) processed in parallel with the samples. Recovery rates for the certified elements were within ± 10% of the reference values, confirming the reliability of the analytical procedure. Additionally, methodological blanks and triplicate analyses were performed during the analytical session.

Average recoveries (Table S1) for each metal(loid) were: 110% (As), 96% (Cd), 111% (Cr), 102% (Cu), 101% (Ni), 100% (Pb), 95% (Zn), 109% (Co), 99% (V), and 103% (Sb). The detection limits (LD) for the analyzed metal(loid)s, expressed in μg L^−1^, were: As = 0.027, Cd = 0.0049, Cr = 0.0226, Cu = 0.0184, Ni = 0.0512, Pb = 0.0047, Zn = 0.178, Co = 0.0010, V = 0.0158, and Sb = 0.0250.

### Soil color and magnetic susceptibility measurements

Color determination in soils is a rapid and non-destructive technique that can serve as a proxy for heavy metal contamination, organic matter content, and iron oxide concentrations. Iron oxides and organic matter may undergo combustion-related transformations into magnetite and carbon. Magnetite, typically black, is commonly associated with anthropogenic heavy metal inputs (Lu et al., 2007; Wang et al., 2019). Soil color was evaluated on fine earth fractions (< 200 µm) using a ColorLite sph860/sph900 spectrophotometer. This device features a 38 mm measurement aperture, a blue and white LED light source, and a 10° observation angle. It records diffuse reflectance spectra with a resolution of 3.5 nm across the 400–700 nm range, which are converted into colorimetric parameters within the CIELAB color space. The spectrophotometer provides objective and sensitive quantification of color, surpassing visual estimates based on standard color charts. The CIELAB system includes the lightness component (L*, ranging from 0 to 100), the a* axis (positive values indicate red hues, negative values green), and the b* axis (positive values indicate yellow, negative values blue) (Viscarra Rossel et al., 2006; Vodyanitskii & Kirillova, 2016).

Magnetic susceptibility (*χ*) of each sample was measured using a Bartington MS2 meter equipped with a dual-frequency MS2B sensor. The instrument applies a low-intensity alternating magnetic field (~ 0.1 mT) and records susceptibility at both low (0.47 kHz) and high (4.7 kHz) frequencies. Low-frequency susceptibility (*χlf*) provides an estimate of the overall concentration of ferrimagnetic minerals in the sample. High-frequency susceptibility (*χhf*), on the other hand, is less influenced by ultrafine superparamagnetic particles, as these particles are unable to respond effectively to high-frequency fields due to their short relaxation times. Frequency-dependent susceptibility (*χfd%*) was also calculated to assess the presence of ultrafine superparamagnetic particles, using the following equation:

1 $$\chi_{fd\% } = \left( {\frac{{\chi_{lf} - \chi_{hf} }}{{\chi_{lf} }}} \right) \times 100$$

High *χfd%* values above 10% are typically interpreted as indicating a significant presence of superparamagnetic particles, often attributed to anthropogenic inputs such as traffic emissions or industrial combustion (Dearing et al., 1996; Maher, 1998).

All measurements were performed in triplicate to reduce instrumental error, and methodological blanks were analyzed to control for contamination. Numerous studies have demonstrated that magnetic susceptibility measurements are effective in detecting industrial emissions and vehicular pollutants in urban environments. Atmospheric deposition is frequently cited as a primary source of magnetic particles and heavy metals in urban soils (Hanesch & Scholger, 2002; Petrovský & Elwood, 1999).

### Contamination assessment

To evaluate the degree of heavy metal contamination and potential ecological risk in urban soils from La Paz, three widely used indices were applied: the Contamination Factor (CF), the Geoaccumulation Index (Igeo), and the Potential Ecological Risk Factor (Er). These indices provide complementary information regarding the enrichment of elements in soils relative to background levels and their potential environmental impact (Hakanson, 1980; Islam et al., 2015; Müller, 1969).

#### Contamination factor (CF)

The CF was calculated to assess the enrichment of a given element relative to local background values using the equation (Hakanson, 1980):

2 $$CF_{i} = \frac{{C_{i} }}{{B_{i} }}$$

where *C*_*i*_ is the concentration of metal *i* in the soil sample and *B*_*i*_ is its corresponding local geochemical background value. CF values are interpreted as: CF < 1: Low contamination; 1 ≤ CF < 3: Moderate contamination; 3 ≤ CF < 6: Considerable contamination; CF ≥ 6: Very high contamination.

#### Geoaccumulation index (Igeo)

The Igeo index was introduced by Müller (1969) to assess metal contamination in sediments and soils. It is defined as:

3 $$Igeo = log_{2} \left( {\frac{{C_{i} }}{{1.5 \times B_{i} }}} \right)$$

where *C*_*i*_ and *B*_*i*_ are as defined above, and the constant 1.5 accounts for natural variability in the background values due to lithogenic effects. The Igeo classification is: Igeo ≤ 0: Uncontaminated; 0 < Igeo ≤ 1: Uncontaminated to moderately contaminated; 1 < Igeo ≤ 2: Moderately contaminated; 2 < Igeo ≤ 3: Moderately to heavily contaminated; 3 < Igeo ≤ 4: Heavily contaminated; 4 < Igeo ≤ 5: Heavily to extremely contaminated; Igeo > 5: Extremely contaminated.

#### Potential ecological risk factor

The potential ecological risk factor (*E*_*r*_) was computed according to Hakanson (1980):

4 $$E_{r}^{i} = T_{r}^{i} \times CF_{i}$$

where *T*_*r*_ is the toxic response factor for each metal. Typical values used in previous studies are: As = 10, Cd = 30, Co = 5, Cr = 2, Cu = 5, Ni = 5, Pb = 5, Sb = 5, V = 2, Zn = 1 (Hakanson, 1980; Islam et al., 2015). The E_r_ values are interpreted as: E_r_ < 40: Low risk; 40 ≤ E_r_ < 80: Moderate risk; 80 ≤ E_r_ < 160: Considerable risk; 160 ≤ E*r* < 320: High risk; E_r_ ≥ 320: Very high risk.

### Spatial analysis

Spatial distribution maps of heavy metal concentrations in surface soils were produced using QGIS (version 3.34, long term release). Sampling locations, georeferenced with a handheld GPS in the WGS 84 coordinate system (EPSG:4326), were imported into the GIS environment and linked to their corresponding analytical data.

The spatial variability of PTEs was modeled using the Inverse Distance Weighting (IDW) interpolation method, a deterministic algorithm that estimates values at unsampled points based on the weighted influence of surrounding samples. IDW parameters (power value, number of neighbors, and search radius) were optimized to capture local gradients while preserving regional patterns, following approaches commonly applied in environmental geochemistry (Guagliardi et al., 2012; Shit et al., 2016).

For visualization, graduated color symbology was applied to represent concentration classes and highlight potential hotspots, facilitating interpretation of spatial patterns and their relationship with land use and anthropogenic activities (Longley et al., 2015; Zhang, 2006). Satellite imagery from Google Maps was used as a base layer for geographic context. This workflow is consistent with standard GIS methodologies for soil contamination assessment (Longley et al., 2015).

### Human health risk assessment

Human health risk assessment was conducted following the guidelines of the United States Environmental Protection Agency (USEPA) to evaluate potential non-carcinogenic risks associated with heavy metals in surface soils (USEPA, 2001). Three main exposure pathways were considered: ingestion of soil particles, inhalation of resuspended soil particles, and dermal contact with contaminated soil. Volatilization exposure was not considered, as the metals studied are not significantly volatile under environmental conditions.

For each pathway, the average daily dose (ADD) was calculated using the following equations:

5 $$ADD_{ing} = \frac{C \times IngR \times EF \times ED}{{BW \times AT_{nc\left( c \right)} }} \times 10^{ - 6}$$

6 $$ADD_{inh} = \frac{C \times InhR \times EF \times ED}{{PEF \times BW \times AT_{nc\left( c \right)} }}$$

7 $$ADD_{der} = \frac{C \times SA \times AF \times ABF \times EF \times ED}{{BW \times AT_{nc\left( c \right)} }} \times 10^{ - 6}$$

Detailed descriptions of the parameters, units, default values, and exposure receptors used in the preceding equations are presented in Table S2.

#### Non-carcinogenic risk

For each exposure pathway, the hazard quotient (HQ) was calculated as:

8 $$HQ = \frac{{ADD_{i} }}{RfD}$$

where RfD is the reference dose specific to each pathway, i.e., oral, inhalation, dermal (Table S3). The hazard index (HI) was then obtained as the sum of the HQ values for all metals and exposure routes:

9 $$HI = \sum HQ_{i}$$

An HI > 1 indicates the potential for non-carcinogenic health risks, whereas HI < 1 indicates no significant risk. The assessment was conducted separately for children and adults, acknowledging the higher sensitivity of children due to behavioral factors and lower body weight (Schiavo et al., 2021).

#### Carcinogenic risk

The carcinogenic risk (CR) was estimated for four metal(loid)s (As, Cr, Ni, and Pb) classified by the International Agency for Research on Cancer (IARC) as Group 1 carcinogens (Kim et al., 2015). The risk for each pathway was calculated using the following equation:

10 $$CR = CDI_{i} \times SF$$

and the total carcinogenic risk (TCR) was obtained as:

11 $$TCR = \sum CR_{i}$$

where SF (mg kg^−1^ day^−1^) is the carcinogenic slope factor provided by the USEPA (2010). Table S3 lists the SF for As, Cr, Ni, and Pb for the three exposure pathways (ingestion, inhalation, and dermal contact). According to the USEPA risk characterization criteria, TCR values > 1 × 10^−4^ are considered intolerable, whereas TCR < 1 × 10^−6^ indicates negligible cancer risk. Intermediate values (10^−6^–10^−4^) are regarded as within the acceptable or tolerable risk range (Dat et al., 2021).

### Statistical analysis

Pearson correlation coefficients (r) were calculated to evaluate linear relationships among PTE concentrations in soils. The statistical significance of each correlation was assessed using *p*-values, with *p* < 0.05 considered significant. A heatmap was generated to visualize the correlation structure, with significant correlations highlighted for interpretation. Furthermore, to identify the main gradients of variation in the dataset, a Principal Component Analysis (PCA) was performed on z-score-standardized concentrations. The first two principal components (PC1 and PC2), which together accounted for the largest proportion of the total variance, were retained for interpretation. All computations and visualizations were performed in Python 3.11 using the pandas, numpy, scipy, scikit-learn, matplotlib, and seaborn libraries.

## Results and discussion

### PTE concentrations

**Fig. 2:**
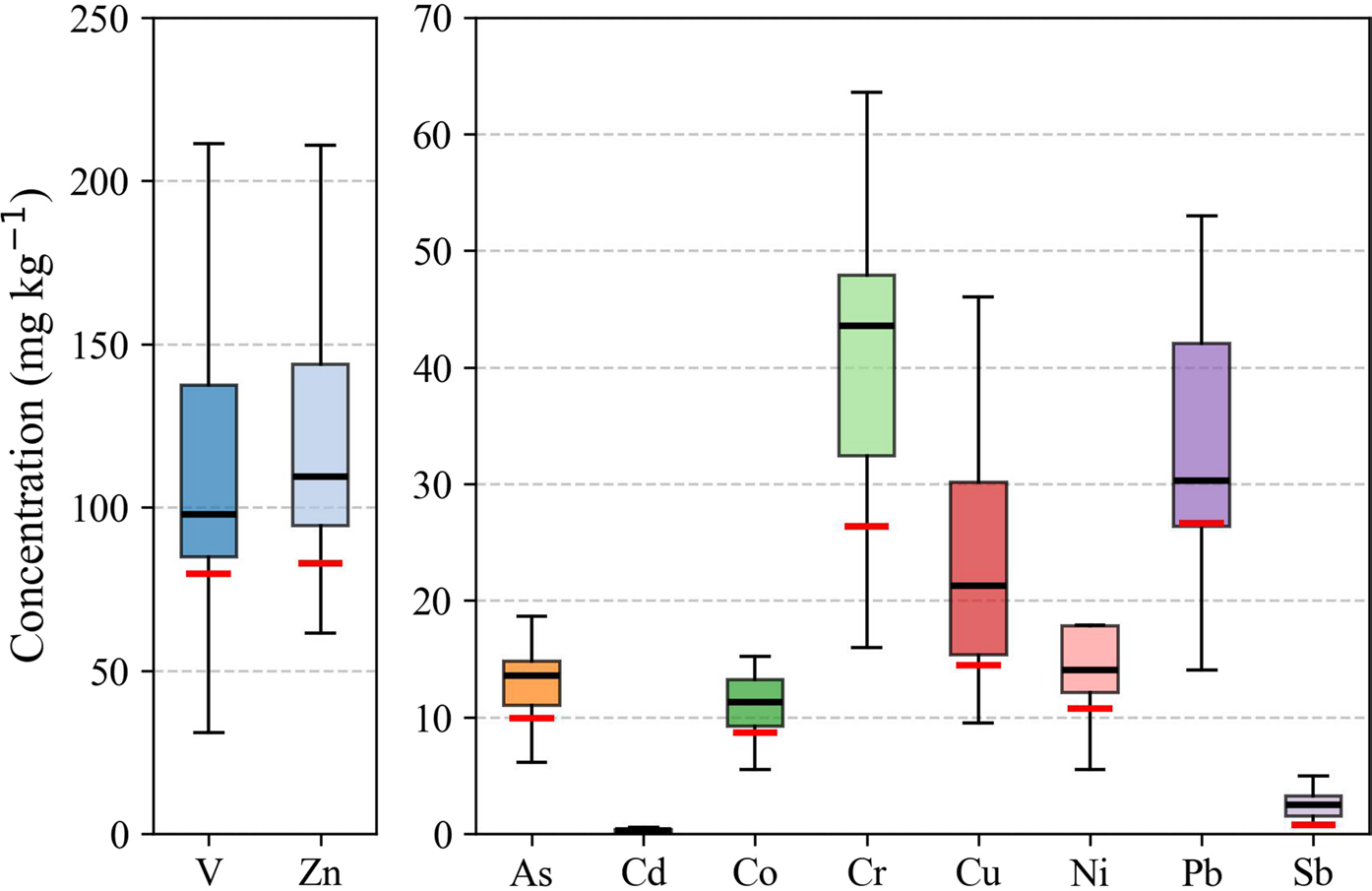
Boxplots showing the distribution of PTE concentrations (mg kg^−1^) in surface soils from La Paz. The black lines represent the median values, the boxes the interquartile range, and whiskers the minimum and maximum values. Red bars indicate background values for each element

Descriptive statistics and visual representations of heavy metal concentrations in the surface soils of La Paz are presented in Fig. 2 and Table 1. The average concentrations (mg kg^−1^) followed the order: Zn (132.37) > V (112.48) > Cr (44.24) > Pb (38.23) > Cu (24.09) > Ni (23.78) > As (12.98) > Co (11.80) > Sb (2.58) > Cd (0.31). The highest individual concentration was observed for Zn (325.95 mg kg^−1^), followed by V (302.80 mg kg^−1^), Pb (142.70 mg kg^−1^), and Ni (129.70 mg kg^−1^). Although Cd exhibited the lowest mean concentration among all elements (0.33 mg kg^−1^), it was consistently detected in all samples, with values well above the instrumental detection limit.

**Table 1:** Concentration (mg kg^−1^) of PTEs analyzed in soils from the urban area of La Paz, highway, and around two power plants. Statistical values and geochemical background levels are also reported

Sample	As	Cd	Co	Cr	Cu	Ni	Pb	Sb	V	Zn
CTPP-1	14.36	0.27	13.1	87.16	30.14	129.7	27.35	3.75	302.8	143.87
CTPP-2	15.94	0.49	9.42	92.48	20.71	14.34	14.05	2.53	94.5	62.53
CTPP-3	23.84	0.88	13.84	71.71	41.95	28.55	31.45	3.17	152.01	210.97
CTPP-4	13.07	0.54	12.8	63.55	26.55	95.29	29.6	3.44	211.4	274.57
CTPP-5	1.48	0.34	8.96	47.56	45.21	47.19	26.37	0.69	188.79	200.85
CTPP-6	13.56	0.25	22.37	46.11	21.29	13.3	24.26	1.74	145.82	100.48
CCI-1	18.58	0.26	15.2	44.51	22.7	17.89	42.86	2.09	135.96	145.63
CCI-2	12.62	0.15	12.29	32.38	13.06	11.68	33.85	5.9	110.36	109.66
CCI-3	12.28	0.18	11.45	35.13	15.75	14.53	35.28	1.4	99.08	117.08
CCI-4	9.98	0.11	10.9	29.16	12.89	9.74	23.13	1.38	87.66	94.46
CCI-5	3.53	0.14	10.98	35.15	15.43	27.32	28.4	1.54	156.2	108.57
CCI-6	12.42	0.19	7.95	23.94	9.5	13.37	28.53	2.67	63.09	71.47
CCI-7	11.55	0.26	9.9	28.06	14.6	11.84	30.27	3.2	46.92	83.68
Highway-CTPP-1	17.89	0.18	8.25	50.35	13.51	14.04	24.27	2.61	88.13	91.6
Highway-CTPP-2	14.48	0.2	19.71	47.9	20.55	14.4	21.92	2.29	137.38	106.64
Highway-CTPP-3	9.73	0.38	11.28	38.64	46	13.71	50.6	1.89	104.83	111.99
Highway-CCI-1	11.01	0.15	10.92	31.44	14.6	12.83	25.72	1.26	88.29	99.28
Highway-CCI-2	9.89	0.21	7.69	35.53	15.33	16.58	28.8	0.96	63.15	87.11
Highway-CCI-3	18.62	0.72	13.22	50.87	43.59	17.85	142.7	2.33	120.51	325.95
Park Arboladas	14.56	0.21	12.86	46.64	24.56	12.13	42.06	3.97	97.79	109.42
Park El Manglito	6.14	0.23	5.53	15.97	17.89	5.51	53	1.3	30.92	61.55
Park Olachea	16.28	0.32	13.97	43.59	35.59	17.15	49.93	4.98	85.57	142.33
Park La Ladrillera	13.72	0.34	9.21	29.74	30.16	12.39	39.25	3.19	50.28	132.82
Park Costa Azul	14.18	0.34	14.25	46.12	25.02	12.04	36.21	3.82	84.89	98.49
Park GV	14.78	0.39	9.07	32.4	25.74	11.06	65.94	2.47	65.77	218.27
Background	9.93	0.10	8.65	26.34	14.45	10.71	26.64	0.76	79.73	82.87
Mean	12.98	0.31	11.80	44.24	24.09	23.78	38.23	2.58	112.48	132.37
Maximum	23.84	0.88	22.37	92.48	46	129.70	142.70	5.90	302.80	325.95
Minimum	1.48	0.11	5.53	15.97	9.50	5.51	14.05	0.69	30.92	61.55
Median	13.56	0.26	11.28	43.59	21.29	14.04	30.27	2.47	97.79	109.42
SD	4.75	0.18	3.68	18.31	10.97	28.33	24.67	1.27	59.01	65.72
Grade I	22	37	nr	nr	nr	1,600	400	nr	78	nr
Grade II	260	450	nr	nr	nr	20,000	800	nr	1000	nr
WHO guideline soil quality limits	nr	0.8	nr	100	36	35	85	nr	nr	50

CTPP, punta prieta thermoelectric power plant; CCI, internal combustion power plant; SD, standard deviation; nr, not reported; grade I, Mexican soil quality guideline for residential, agricultural, and commercial areas; grade II, for industrial areas; grade III, world health organization (WHO) soil quality guideline

When compared with local geochemical background levels, a clear enrichment of heavy metals was observed across the soil samples (Fig. 2), suggesting the influence of anthropogenic activities such as industrial emissions, vehicular traffic, and atmospheric deposition. On a sample-by-sample basis, Cd concentrations exceeded background levels in 100% of the sites, followed closely by Sb (96%), Cr and Ni (92%), Zn (88%), Co and Cu (84%), As (80%), and V and Pb (76%). These high exceedance frequencies underscore a widespread distribution of contaminants across the urban and industrial landscape. In terms of magnitude, the mean concentrations were 2.5 times higher for Cd, 2.1 for Zn, and 1.4 for Pb compared to background values. Other metals also showed substantial enrichments: Sb was enriched by a factor of 2.8, Cr by 1.9, Cu by 1.6, Ni by 1.5, Co by 1.4, and As by 1.3. These levels reflect diffuse but significant metal loading across the urban environment. This pattern is consistent with previous findings in urban-industrial environments, where non-ferrous metals and fossil fuel combustion residues accumulate in surface soils due to poor atmospheric dispersion and proximity to emission sources (Maxim et al., 2022). The magnitude and frequency of exceedance collectively indicate that metal inputs in the region are not isolated but rather pervasive, and they warrant continued monitoring and source identification efforts.

To assess potential health and environmental implications, PTE concentrations were compared with both the Mexican soil quality guideline NOM-147-SEMARNAT/SSA1-2004 (SEMARNAT & SSA, 2007) and the World Health Organization (WHO, 1994) soil quality guideline. For urban parks, areas intended for residential, recreational, or agricultural use, concentrations were evaluated against Grade I thresholds and WHO values when available. Overall, all PTEs remained below their respective Grade I limits except vanadium. The mean V concentration (69.20 mg kg^−1^) approached the Grade I threshold (78 mg kg^−1^), although still below the WHO guideline (100 mg kg^−1^). In contrast, As (13.28 mg kg^−1^), Cd (0.31 mg kg^−1^), Pb (47.73 mg kg^−1^), and Ni (11.71 mg kg^−1^) were all well below their Grade I limits (22, 37, 400, and 1600 mg kg^−1^, respectively) and also below the corresponding WHO values for Cd (0.8 mg kg^−1^), Cr (100 mg kg^−1^), Pb (85 mg kg^−1^), Ni (35 mg kg^−1^), and Zn (50 mg kg^−1^). These results indicate that urban soils generally comply with both national and international quality standards, although vanadium concentrations warrant continued monitoring due to their proximity to regulatory limits. For soils collected near the thermoelectric power plants (CTPP and CCI), comparisons were made with Grade II limits, applicable to industrial land use, as well as WHO guidelines where available. All PTEs were within permissible Grade II limits. Arsenic concentrations at CTPP (13.71 mg kg^−1^) and CCI (11.57 mg kg^−1^) were well below the Grade II limit (260 mg kg^−1^), although no WHO reference value exists for As. Cd levels (0.46 mg kg^−1^ at CTPP and 0.18 mg kg^−1^ at CCI) were substantially lower than both the Grade II limit (450 mg kg^−1^) and the WHO threshold (0.8 mg kg^−1^). Pb concentrations (25.51 mg kg^−1^ at CTPP and 31.76 mg kg^−1^ at CCI) remained far below the industrial limit (800 mg kg^−1^) and the WHO value (85 mg kg^−1^). Ni concentrations (54.73 mg kg^−1^ at CTPP; 15.20 mg kg^−1^ at CCI) were well below the Grade II limit (20,000 mg kg^−1^) and also below the WHO guideline (35 mg kg^−1^). Finally, Zn levels remained under both national and WHO thresholds. These findings show that although industrial activity contributes to elevated local metal loads, particularly for V and Ni, soil concentrations around the power plants remain within acceptable limits for industrial land use and do not exceed the available WHO guidelines.

### Site-based concentration patterns

**Fig. 3:**
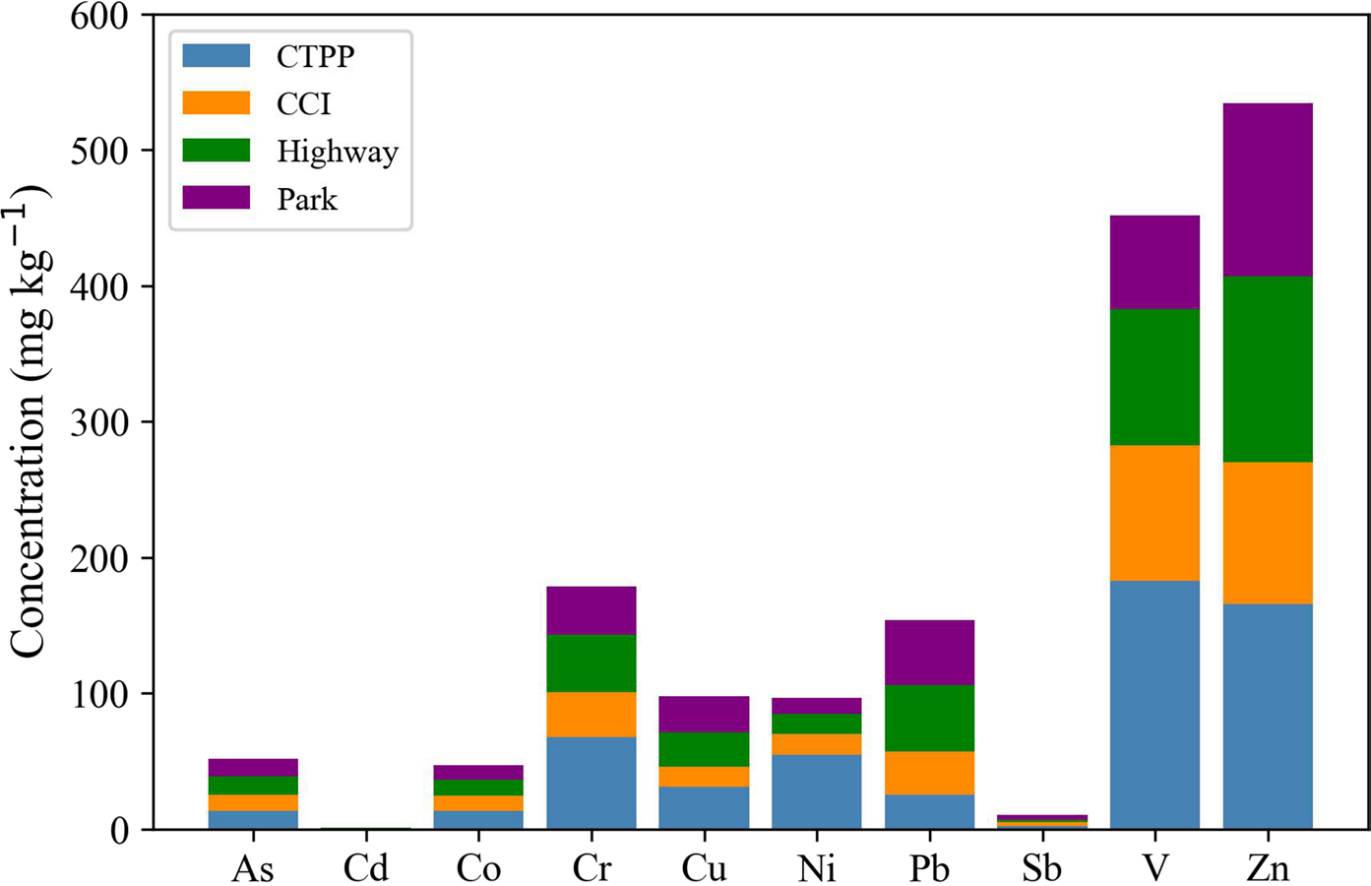
Stacked bar chart of mean concentrations (mg kg⁻^1^) of PTEs in soils from the four sampling site categories: punta prieta thermoelectric power Plant (CTPP), internal combustion power plant (CCI), Highway, and Park. The height of each colored segment represents the contribution of each site type to the total concentration of the corresponding element

PTE concentrations varied markedly depending on the sampling site, reflecting distinct anthropogenic inputs and land use characteristics across the study area (Fig. 3 and Table S4). Soils collected near the CTPP exhibited the highest average concentrations for most PTEs, including Cr (68.10 mg kg^−1^), Ni (54.73 mg kg^−1^), V (182.55 mg kg^−1^), and Zn (165.55 mg kg^−1^). These elevated values are consistent with emissions from heavy fuel oil combustion and industrial processes, which are known to enrich soils with V and Ni through atmospheric deposition (Vishnyakov, 2023). The high variability of Ni (SD = 47.66 mg kg^−1^) further suggests uneven deposition patterns and possible influence from legacy contamination. In contrast, soils near the CCI presented overall lower concentrations, although Pb (31.76 mg kg^−1^) and Sb (2.60 mg kg^−1^) were notable, likely influenced by emissions and traffic associated with the industrial corridor. Along the highway corridor, Pb (49.00 mg kg^−1^) reached its highest average value, while Sb (1.89 mg kg^−1^) and Zn (137.10 mg kg^−1^) also showed elevated levels, reflecting the impact of heavy vehicular activity. The wide range and standard deviation observed for Pb (SD = 47.07 mg kg^−1^) suggest localized hotspots, potentially linked to brake wear, fuel residues, and road dust accumulation. Soils from urban parks, although generally assumed to be less impacted, displayed elevated concentrations of certain elements, particularly Sb (3.29 mg kg^−1^), the highest among all groups, and Pb (47.73 mg kg^−1^). This is likely attributable to their location within residential neighborhoods adjacent to streets with consistent local traffic. Given that Sb is a well-established traffic-related tracer, primarily originating from brake pads and tire abrasion (Parviainen et al., 2020), its presence in park soils underscores the pervasive influence of vehicular emissions even in semi-protected urban spaces.

Overall, these patterns reveal a complex spatial distribution of PTEs driven by both point sources (e.g., power plants) and diffuse sources (e.g., urban traffic), highlighting the importance of land-use context in interpreting contamination profiles and guiding risk mitigation strategies. The simultaneous influence of power plant emissions, combustion by-products, and vehicular-derived particles results in overlapping contamination signatures across sites, complicating source attribution and underscoring the need for integrated monitoring frameworks. Additionally, the presence of traffic-related metals such as Sb and Pb in soils from residential park areas emphasizes that no urban zone is entirely exempt from contamination pressures, even in the absence of direct industrial activity. This spatial heterogeneity must be considered when establishing regulatory thresholds, prioritizing remediation efforts, and developing sustainable land-use planning in rapidly expanding urban environments.

### Comparison between industrial areas

The mean concentrations of PTEs in soils near the CTPP and the CCI in La Paz, Mexico, were compared with values reported for soils surrounding various fuel-based power plants worldwide (Table 2).

**Table 2:** Comparison of mean concentrations (mg kg^−1^) of PTEs in soils from La Paz, Mexico with those reported in areas influenced by different types of fuel-based power plants worldwide

City and country	Fuel type	As	Cd	Co	Cr	Cu	Ni	Pb	Sb	V	Zn
La Paz, Mexico (CTPP)^a^	Oil	13.71	0.46	13.42	68.10	30.98	54.73	25.51	2.55	182.55	165.55
La Paz, Mexico (CCI)^a^	Oil and diesel	11.57	0.18	11.24	32.62	14.85	15.20	31.76	2.60	99.90	104.36
Panzhihua, China^b^	Coal	50.67	4.45	–	61.70	25.21	–	77.77	–	91.51	699.35
Rosarito, Mexico^c^	Natural gas	–	0.23	–	33	12	48	5.3	–	29	–
Jinsha, China^d^	Coal	19.20	0.60	–	42.14	31.74	–	39.84	–	–	–
Kangal, Turkey^e^	Coal	9	–	63.9	713.20	28.8	610.1	17	–	–	81.8
Kahramanmaras, Turkey^f^	Lignite	–	–	–	191.73	38.50	189.84	–	–	–	102.18
Gacko, Bosnia and Herzegovina^g^	Coal	–	–	19.25	60.23	46.27	82.10	38.70	–	90	109.50
Kütahya, Turkey^h^	Coal	28.1	0.11	–	288	19.6	558	21.1	–	–	57.9
Xilin Gol, Mongolia^i^	Coal	25.13	1.11	–	–	12.42	–	5.67	–	–	23.21

^a^This work; ^b^Long et al. (2021); ^c^Pastrana-Corral et al. (2017); ^d^Huang et al. (2017); ^e^Turhan et al. (2020); ^f^Akbay et al. (2023); ^g^Ilić et al. (2022); ^h^Özkul, (2016); ^i^Zhang et al. (2020);

At CTPP, V showed the highest enrichment (182.55 mg kg^−1^), clearly exceeding values reported for several coal-fired settings such as Panzhihua, China (91.51 mg kg^−1^; Long et al., 2021) and Gacko, Bosnia and Herzegovina (90 mg kg^−1^; Ilić et al., 2022), and far above the natural-gas thermal power plant at Rosarito, Mexico (29 mg kg^−1^; Pastrana-Corral et al., 2017). Ni at CTPP (54.73 mg kg^−1^) was below the exceptionally high levels reported for coal plants in Turkey (Kangal: 610.1 mg kg^−1^, Turhan et al., 2020; Kütahya: 558 mg kg^−1^, Özkul, 2016), but higher than at Rosarito (48 mg kg^−1^; Pastrana-Corral et al., 2017) and comparable to several coal sites with moderate Ni (e.g., Gacko: 82.10 mg kg^−1^; Ilić et al., 2022). Cr near CTPP (68.10 mg kg^−1^) was greater than Jinsha, China (42.14 mg kg^−1^; Huang et al., 2017) and Panzhihua (61.70 mg kg^−1^; Long et al., 2021), but far below Kangal (713.20 mg kg^−1^; Turhan et al., 2020) or Kahramanmaraş lignite (191.73 mg kg^−1^; Akbay et al., 2023). Cd at CTPP (0.46 mg kg^−1^) was modest compared with Panzhihua (4.45 mg kg^−1^; Long et al., 2021) and close to Jinsha (0.60 mg kg^−1^; Huang et al., 2017). For Zn, CTPP (165.55 mg kg^−1^) was well below the extreme value at Panzhihua (699.35 mg kg^−1^; Long et al., 2021), but higher than several other coal sites (e.g., Kütahya: 57.9 mg kg^−1^, Özkul, 2016; Gacko: 109.50 mg kg^−1^, Ilić et al., 2022; Xilin Gol: 23.21 mg kg^−1^, Zhang et al., 2020) and comparable to Kahramanmaraş (102.18 mg kg^−1^; Akbay et al., 2023). Co at CTPP (13.42 mg kg^−1^) fell between the lower value at Gacko (19.25 mg kg^−1^; Ilić et al., 2022) and the much higher level at Kangal (63.9 mg kg^−1^; Turhan et al., 2020). Pb at CTPP (25.51 mg kg^−1^) was below Panzhihua (77.77 mg kg^−1^; Long et al., 2021) and similar to Gacko (38.70 mg kg^−1^; Ilić et al., 2022). In contrast, at CCI, metals were consistently lower, reflecting cleaner fuel use. V (99.90 mg kg^−1^) was about half the CTPP level yet still above Rosarito’s natural-gas site (29 mg kg^−1^; Pastrana-Corral et al., 2017). Ni (15.20 mg kg^−1^) and Cr (32.62 mg kg^−1^) were low relative to coal plants (e.g., Kangal and Kütahya for Ni and Cr; Turhan et al., 2020 and Özkul, 2016) and comparable to Jinsha (Cr = 42.14 mg kg^−1^; Huang et al., 2017). Cd (0.18 mg kg^−1^) and Pb (31.76 mg kg^−1^) at CCI were also modest when contrasted with coal-affected soils (e.g., Panzhihua Cd = 4.45, Pb = 77.77 mg kg^−1^; Long et al., 2021). Zn at CCI (104.36 mg kg^−1^) was intermediate, higher than Xilin Gol (23.21 mg kg^−1^; Zhang et al., 2020) and similar to Kahramanmaraş (102.18 mg kg^−1^; Akbay et al., 2023). For Co, CCI (11.24 mg kg^−1^) was lower than Gacko (19.25 mg kg^−1^; Ilić et al., 2022) and far below Kangal (63.9 mg kg^−1^; Turhan et al., 2020).

These comparisons confirm the influence of fuel type on soil metal contamination profiles. Heavy fuel oil combustion at CTPP leads to substantial V and Ni enrichment, characteristic of high-sulfur residual fuels. Coal-fired plants elsewhere generally show higher absolute levels of multiple metals (e.g., Ni, Cr, Co, Pb), reflecting both combustion emissions and ash deposition. Natural gas facilities, like thermal power plant of Rosarito, tend to produce significantly lower PTE burdens, although oil/diesel co-firing energy production industry can elevate specific elements such as V and Ni. Overall, the La Paz data align with global trends showing that fuel composition and combustion technology strongly control the magnitude and type of PTE deposition in surrounding soils. The relatively high V and Ni levels near CTPP warrant continued monitoring, given their persistence and potential ecological and human health implications.

### Soil color and magnetic susceptibility

#### Soil color characteristics in surface soil

Soil color parameters measured in the CIELAB space (L*, a*, b*) revealed notable differences across the four sampling categories (Table 3). Lightness values (L*) ranged from 50.97 to 72.70, with lower L* values indicating darker soils potentially enriched in organic matter, combustion residues, or magnetic minerals. The darkest samples were recorded at CCI_4 (L* = 50.97), Highway-CTPP-2 (L* = 53.50), and Highway-CCI-2 (L* = 54.63), suggesting localized accumulation of fine particles and combustion-derived material. In contrast, samples such as CTPP_2 (L* = 70.78) and CCI_2 (L* = 72.70) exhibited the highest lightness, indicating relatively lower content of particulate contaminants or mineral coatings. The a* values (green–red axis) were generally positive across all samples, with values ranging from 3.65 to 12.20, indicating a shift toward reddish hues commonly associated with iron oxides such as hematite and goethite. The b* values (blue–yellow axis) ranged from 11.15 to 23.47, reflecting yellowish hues that also point to Fe oxide influence. Soils from CTPP showed consistently high b* values (mean ≈ 21.7), aligning with known contributions of Fe-rich particles from heavy fuel oil combustion. However, in an urban-industrial context, additional PTEs such as Cu, Ni, Cr, Pb, Sb, and Zn, often bound to fine combustion residues or road dust, can also influence soil coloration. These particles may darken the soil surface (reducing L*), mask underlying mineral colors, or subtly shift chromatic values, particularly in areas with high vehicular or industrial emissions. Among the sampling sites, CTPP soils generally exhibited intermediate to low L*, high a* and b* values, consistent with combined contributions from industrial combustion and deposition of metal-bearing particulates. Highway samples displayed high variability in L*, reflecting heterogeneity in traffic-related deposition and surface conditions. Notably, urban park soils, despite their recreational use, exhibited color patterns comparable to industrial and traffic-impacted zones, including dark to intermediate L* values (mean ≈ 62.3) and elevated a* and b* values, particularly in Park Ladrillera, suggesting inputs from vehicular traffic, atmospheric deposition, and potentially Fe- and metal-rich particulates.

**Table 3:** CIELAB color parameters (L, a*, b*) of surface soil samples collected from four site categories in CTPP, CCI, Highway corridors, and urban parks. Color parameters were measured on fine-earth fractions (< 200 μm) using a spectrophotometer in reflectance mode. L* indicates lightness (0 = black, 100 = white), a* represents the green–red axis, and b* the blue–yellow axis

Sample	L*	a*	b*
CTPP-1	56.56	6.16	18.1
CTPP-2	70.78	7.3	23.47
CTPP-3	64.31	6.91	21.18
CTPP-4	66.3	7.78	22.19
CTPP-5	61.5	7.43	21.68
CTPP-6	59.15	9.25	19.7
CCI-1	57.51	8.58	13.3
CCI-2	72.7	6.54	15.57
CCI-3	62.33	8.82	20.14
CCI-4	50.97	6.06	12.64
CCI-5	54.33	7.31	15.72
CCI-6	63.45	3.65	11.52
CCI-7	63.45	3.65	11.52
Highway-CTPP-1	62.02	8.4	14.09
Highway-CTPP-2	53.5	6.32	11.15
Highway-CTPP-3	61.01	6.28	17.66
Highway-CCI-1	63.39	7.88	23.02
Highway-CCI-2	54.63	6.61	16.59
Highway-CCI-3	63.06	6.18	18.14
Park Arboledas	60.58	6.78	17.94
Park El Manglito	65.9	6.48	19.96
Park Olachea	66.06	6.9	20.72
Park Ladrillera	61.38	12.2	22.28
Park Costa Azul	61.71	6.93	17.63
Park GV	65.99	4.98	14.55

These colorimetric signatures, especially darkening and red–yellow shifts, have been reported as indicative of particulate accumulation rich in Fe oxides and PTEs in soils affected by industrial and urban pollution (Fontes & Carvalho, 2005; Othman et al., 2017). Given the agreement with chemical and magnetic data, color measurements serve as a rapid, non-destructive proxy for assessing contamination and inferring soil pollution sources in urban environments.

#### Magnetic susceptibility in surface soil

Magnetic susceptibility parameters for all sampling sites are presented in Table 4. Supporting sample preparation data, including cube volume, mass, and bulk sample weight, are provided in Table S5. Bulk density (*ρ*) values ranged from 1.36 × 10^3^ to 1.79 × 10^3^ kg m^−3^, with no clear spatial pattern, although slightly higher densities were observed in some urban park and highway samples.

**Table 4:** Magnetic susceptibility parameters of surface soil samples from four site categories in La Paz: CTPP, CCI, Highway corridors, and urban parks

Sample	Klf (SI)	Khf (SI)	Χlf (m^3^ kg^−1^)	Χhf (m^3^ kg^−1^)	Χfd (%)
CTPP_1	0.0347	0.0344	2.46E-05	2.44E-05	0.86
CTPP_2	0.039	0.0389	2.60E-05	2.60E-05	0.26
CTPP_3	0.0157	0.0149	1.01E-05	9.61E-06	5.10
CTPP_4	0.035	0.0345	2.57E-05	2.53E-05	1.43
CTPP_5	0.0342	0.034	2.17E-05	2.16E-05	0.58
CTPP_6	0.0655	0.0657	3.92E-05	3.915E-05	0.30
CCI_1	0.0074	0.0069	4.79E-06	4.47E-06	6.76
CCI_2	0.0138	0.0134	8.29E-06	8.05E-06	2.90
CCI_3	0.004	0.0038	2.91E-06	2.76E-06	5.00
CCI_4	0.0072	0.0068	5.18E-06	4.89E-06	5.56
CCI_5	0.0082	0.0077	5.35E-06	5.02E-06	6.10
CCI_6	0.0081	0.0078	5.03E-06	4.85E-06	3.70
CCI_7	0.0108	0.0103	6.59E-06	6.29E-06	4.63
Highway-CTPP-1	0.0198	0.0191	1.37E-05	1.32E-05	3.54
Highway-CTPP-2	0.0858	0.0856	5.66E-05	5.65E-05	0.23
Highway-CTPP-3	0.0256	0.025	1.73E-05	1.69E-05	2.34
Highway-CCI-1	0.0095	0.0089	5.49E-06	5.14E-06	6.32
Highway-CCI-2	0.011	0.0104	6.71E-06	6.35E-06	5.45
Highway-CCI-3	0.0318	0.0311	1.84E-05	1.80E-05	2.20
Park arboledas	0.0174	0.0163	1.11E-05	1.04E-05	6.32
Park el manglito	0.0169	0.0159	1.09E-05	1.03E-05	5.92
Park olachea	0.0156	0.0146	8.76E-06	8.20E-06	6.41
Park ladrillera	0.0168	0.0158	1.05E-05	9.84E-06	5.95
Park costa azul	0.0197	0.0188	1.22E-05	1.16E-05	4.57
Park GV	0.0303	0.0293	1.70E-05	1.64E-05	3.30
Background	0.0163	0.0153	1.05E-05	9.82E-06	6.13

Klf, volumetric magnetic susceptibility (SI) at low frequency; Khf, volumetric magnetic susceptibility (SI) at high frequency; χlf mass-specific magnetic susceptibility (m^3^ kg^−1^) at low frequency; χhf, mass-specific magnetic susceptibility (m^3^ kg^−1^) at high frequency, χfd, frequency-dependent magnetic susceptibility (%)

Magnetic susceptibility (χlf) values in the surface soils of the study area range from 3 × 10^−6^ to 5.7 × 10^−5^ m^3^ kg^−1^, with a mean of 1.5 × 10^−5^ m^3^ kg^−1^. The lowest concentrations were noted at the background site, outside the direct influence of urban and industrial activities, and unexpectedly low values were also observed in soils near the CCI. In contrast, the highest χlf values were measured around the CTPP and along major traffic corridors. This spatial distribution suggests that while CTPP and traffic emissions are significant contributors of ferrimagnetic particles to urban soils, the CCI plant appears to contribute less to the magnetic signal, possibly due to differences in emission controls, fuel type, or local depositional conditions. Urban Park soils displayed intermediate χlf values but, in some cases (e.g., Park Ladrillera and GV), exceeded those at CCI sites, suggesting that even recreational areas in residential zones are subject to atmospheric deposition of ferrimagnetic particles from nearby traffic corridors (Wang et al., 2019). These patterns align with previous studies linking elevated χlf in roadside and industrial soils to anthropogenic magnetic particle inputs (Lu et al., 2007). χfd in study site surface soils ranges from 0.23 to 6.76% (mean = 3.9%). No values above 10% were detected, indicating a limited contribution of superparamagnetic (SP) grains typically generated by pedogenic processes. The highest χfd% values, between 5.1 and 6.8%, were recorded at the background site and in several samples collected around the CCI, suggesting a relatively higher contribution of fine-grained SP particles in these locations. In contrast, the lowest values, typically below 2% (0.23–1.4%), were observed in soils near the CTPP and along major traffic corridors, where the magnetic signal is dominated by coarser ferrimagnetic grains of anthropogenic origin produced by fossil fuel combustion and industrial emissions. Intermediate values (2–5%) occurred mainly in recreational and mixed-use urban areas, reflecting a combination of natural pedogenic input and atmospheric deposition of technogenic dust. This spatial distribution of χfd% is consistent with previous studies in urban soil, sediments, and roads near industrialized areas (Chrysakopoulou et al., 2025; Lu et al., 2007; Wei & Yang, 2010).

The contrast between industrial/urban hotspots and the background site demonstrates the sensitivity of magnetic parameters as a proxy for detecting the impact of energy production and traffic-related activities on La Paz soils. The integration of magnetic and geochemical data thus provides a robust framework for identifying contamination sources and assessing their spatial footprint.

### Pollution assessment

**Fig. 4:**
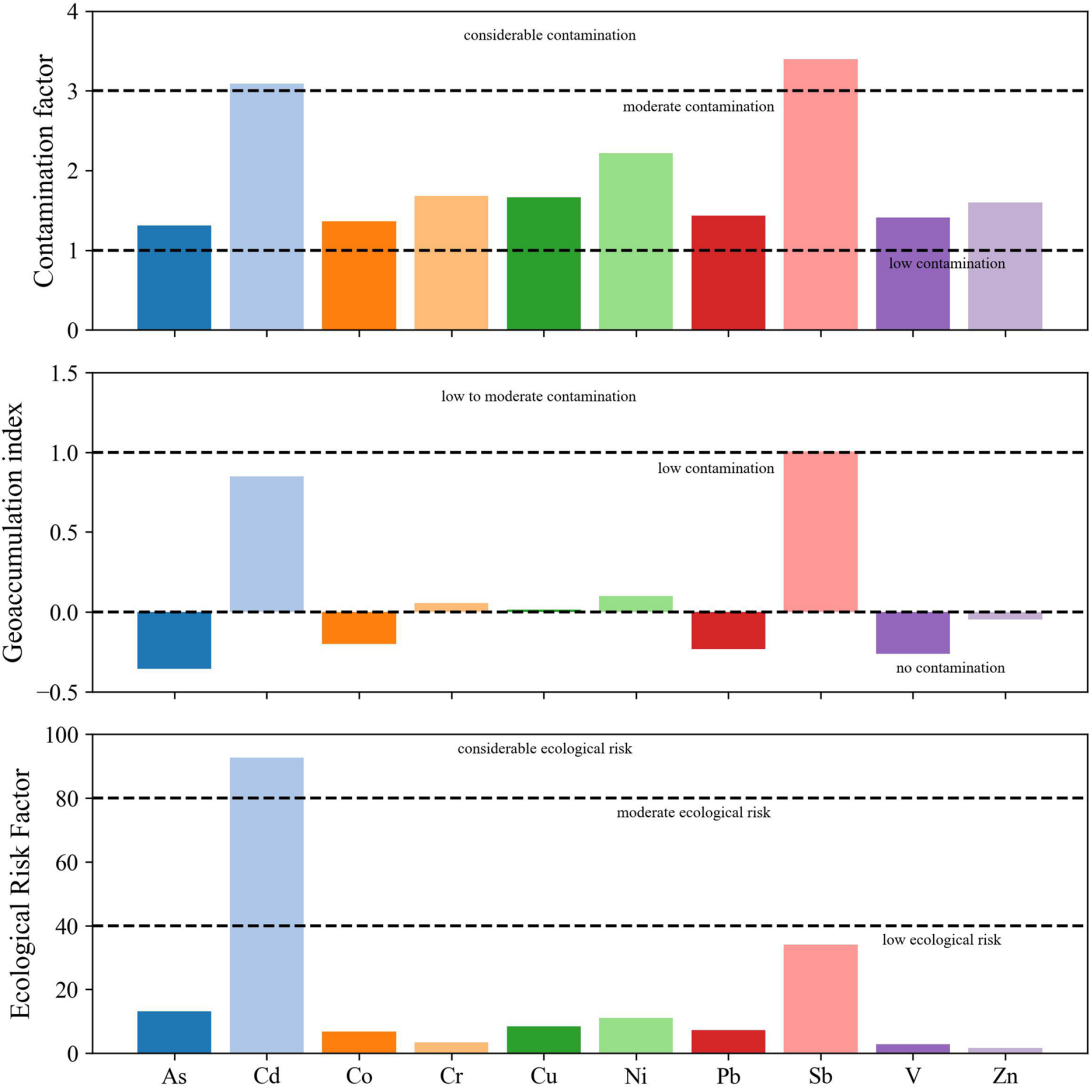
Contamination factor (CF), Geoaccumulation Index (Igeo), and Ecological Risk Factor (Er) for the analyzed PTEs in La Paz soils. Dashed horizontal lines represent contamination and ecological risk thresholds

To assess the level of soil contamination in the study area, three commonly used indices were employed: the CF, Igeo, and Er. These indices help to differentiate between natural and anthropogenic contributions and to determine the degree of ecological hazard posed by PTEs in soils. The results of the pollution indices are presented in Fig. 4 and Table S6. The CF values revealed considerable variability among the analyzed elements. The highest mean CF was observed for antimony (Sb = 3.40), followed by cadmium (Cd = 3.09) and nickel (Ni = 2.22), suggesting moderate to considerable contamination levels. In contrast, vanadium (V = 1.41), cobalt (Co = 1.36), and lead (Pb = 1.44) exhibited moderate contamination, while arsenic (As = 1.31), chromium (Cr = 1.68), copper (Cu = 1.67), and zinc (Zn = 1.60) also fell within moderate contamination thresholds. Cd stood out with a maximum CF value of 8.80, far exceeding the threshold for considerable contamination (CF > 3), indicating severe localized enrichment likely linked to anthropogenic activities such as industrial emissions or improper disposal of industrial waste. Similarly, Sb displayed both a high mean and maximum CF (up to 7.76), further supporting its classification as a contaminant of concern in this environment. These values are consistent with findings in other industrial areas, where Sb and Cd have been reported at elevated levels near smelting, energy generation, or transportation sources (Long et al., 2021; Xu et al., 2021).

The Igeo values provide insight into the extent of anthropogenic influence compared to background concentrations. Most elements exhibited negative or near-zero Igeo means, suggesting background to low contamination. However, Sb (mean = 1.00) and Cd (mean = 0.85) showed elevated Igeo values, falling within the “uncontaminated to moderately contaminated” category according to Müller’s classification. The maximum Igeo values for Sb (2.37) and Cd (2.55) indicate “moderately to strongly contaminated” conditions at specific sampling sites. These findings imply a localized accumulation of Cd and Sb likely resulting from industrial processes or atmospheric deposition from nearby power plants and traffic emissions. Conversely, elements such as (mean =  − 0.36), Co (− 0.20), and V (− 0.26) presented Igeo values within the “uncontaminated” range, reinforcing the inference that their presence in soils is primarily geogenic. Anyway, considering the previously published works by Ganor et al. (1988) and Khan et al. (2011), soils in the vicinity of thermal power plants tend to accumulate V, which, as reported, concentrates predominantly near the emission sources due to atmospheric deposition of particulate matter. Further details on the spatial behavior and distribution of V can be found in Sect. “Spatial distribution”.

The Er incorporates both the contamination level and the toxic response factor of each metal, offering a more risk-weighted evaluation. Cd again emerged as the most hazardous element, with a mean Er of 92.76 and a maximum value of 264, which exceeds the threshold for considerable ecological risk (Er > 80). Sb followed with a mean Er of 33.98, classified as low to moderate ecological risk. Other elements, including As (13.09), Pb (7.18), and Ni (11.10), showed low ecological risk, while Cr, Cu, and Co displayed very low Er values. Zn and V had the lowest mean Er values (1.60 and 2.82, respectively), confirming their minimal contribution to potential ecological harm. Overall, the results emphasize that Cd and Sb are the primary ecological concerns in the study area, due to their high bioavailability and toxicity. These metals require attention in future monitoring and mitigation strategies. The elevated Er values for Cd, in particular, align with global studies that have identified Cd as a high-risk pollutant in urban-industrial settings (Lin et al., 2015; Wang et al., 2015).

### Spatial distribution

**Fig. 5:**
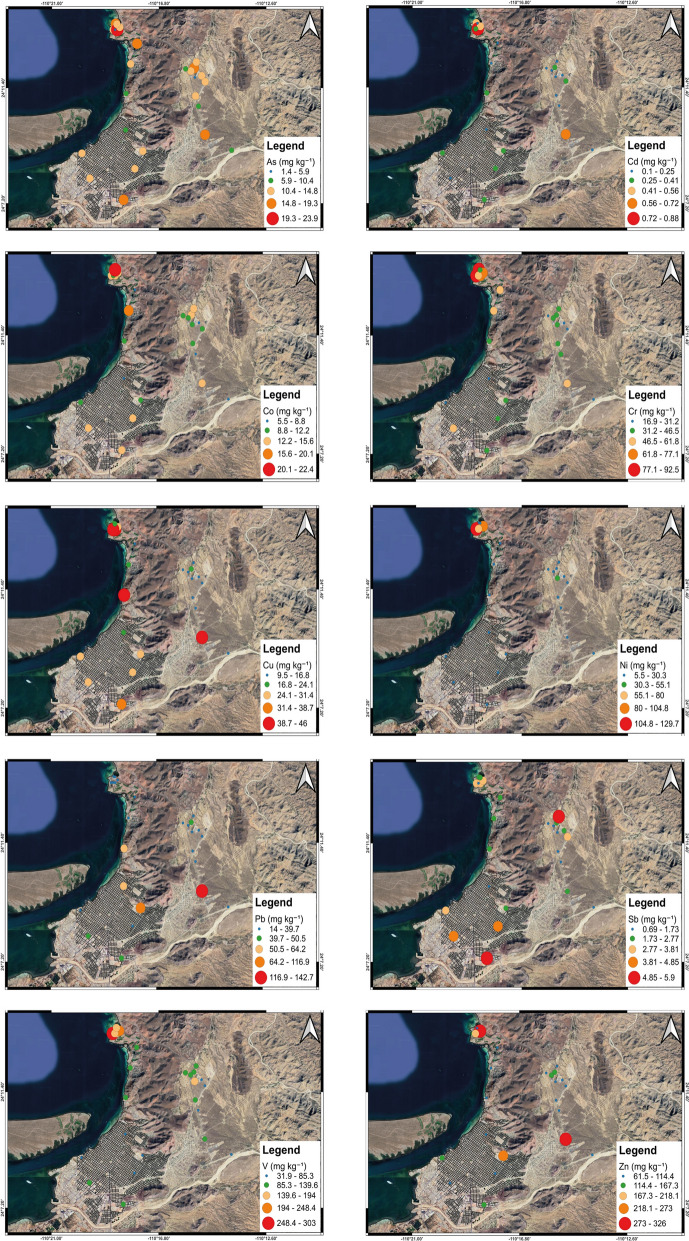
Spatial distribution maps of PTEs (As, Cd, Co, Cr, Cu, Ni, Pb, Sb, V, Zn) in surface soils of La Paz. Warmer colors indicate higher concentrations, with hotspots located near industrial point sources and major traffic corridors

The spatial distribution maps of PTE concentrations (Fig. 5) reveal distinct patterns linked to both point and diffuse emission sources in La Paz. The highest concentrations for several elements were observed in the northern sector of the study area, particularly near the CTPP and, to a lesser extent, the CCI. This area coincides with the principal industrial emissions zone, where heavy fuel oil combustion produces emissions enriched in V, Ni, and other PTEs.

V and Ni show the clearest industrial signature, with maximum values recorded in soils adjacent to CTPP. These patterns are consistent with the well-documented role of V and Ni as markers of residual oil combustion (Khan et al., 2011; Vishnyakov, 2023). Elevated values also occur along the highway connecting CTPP to the urban core, indicating the combined effects of industrial plume deposition and vehicular traffic (Chacón-Patiño et al., 2022). Sb concentrations are markedly higher along major roadways, especially near the highway sites adjacent to both power plants. Given its recognized association with brake and tire wear, Sb serves as a reliable tracer of traffic-related emissions. Urban parks in residential areas with nearby streets also exhibit detectable Sb enrichment, suggesting atmospheric deposition from local traffic sources (Földi et al., 2018). Zn and Cu show intermediate to high values in both industrial and urban roadside areas. Their distribution likely reflects multiple inputs: industrial emissions, vehicle-related wear (tires, brake pads), and, in some cases, urban dust re-suspension. Elevated Zn levels in particular are evident at CTPP and along the coastal highway, aligning with the prevailing wind direction from the north. Cr, Pb, and Co display more heterogeneous patterns, with localized hotspots near both CTPP and CCI, as well as in certain urban park sites. These distributions suggest contributions from both industrial activity and urban sources, potentially including historical contamination and construction materials. Cd concentrations, although low in absolute terms, are elevated relative to background across all site categories, indicating diffuse deposition. The spatial maps show no sharp hotspots but a consistent enrichment near industrial zones and along the highway. Finally, As presents a relatively uniform spatial pattern, with slightly higher concentrations at certain park sites and near CTPP. This distribution may reflect a combination of natural geochemical background and minor anthropogenic inputs.

Overall, the spatial data indicate that point sources, particularly CTPP and to a lesser extent CCI, control the distribution of V and Ni. These emissions strongly affect soils in their immediate surroundings. Instead, vehicular traffic is a major diffuse source for Sb, Zn, and Cu, with contamination signals extending into residential park areas. Several PTEs, such as Cr, Pb, Co, and As, show mixed spatial signatures, reflecting contributions from both industrial and urban activities. Prevailing winds from the north-northwest likely transport industrial particulates toward central and southern La Paz, enhancing their dispersion and contributing to soil pollution. Integrating spatial geochemical patterns with source-specific tracers is essential to distinguish between industrial and traffic-related contamination. This approach also helps identify priority areas for targeted mitigation.

### Source appointment of PTEs

To complement the spatial observations and improve source identification, Pearson correlation analysis and principal component analysis (PCA) were applied to the PTE dataset. These multivariate approaches allow the identification of element associations that may reflect common sources or similar environmental behavior.

Pearson correlation analysis (Fig. S2 and Table 5) revealed several strong and significant associations among the analyzed elements, indicating potential common sources or similar transport and deposition mechanisms. The strongest correlation was between Ni and V (r = 0.87, *p* < 0.01), suggesting a close linkage typically associated with oil combustion in power plants. Other strong correlations included Cd–Cu (r = 0.71, *p* < 0.01), Cd–Zn (r = 0.71, *p* < 0.01), Cu–Zn (r = 0.64, *p* < 0.01), and Cr–V (r = 0.67, *p* < 0.01). These associations are consistent with known emission profiles, where Cd–Cu–Zn often originate from vehicular wear and certain industrial processes, while Cr–V is linked to combustion residues and metallurgical activities. Moderate but significant correlations, such as Pb–Zn (r = 0.63, *p* < 0.01) and As–Cd (r = 0.53, *p* < 0.01), point to overlapping influences from both urban and industrial sources. The correlation between As and Sb (r = 0.47, *p* < 0.05) may reflect a mixed geogenic–anthropogenic signal, given Sb’s role as a traffic tracer and As’s partial geogenic origin in local soils. These correlation patterns, when considered alongside the spatial distribution results, indicate that some metals (Ni, V, Cr) are primarily influenced by industrial emissions, while others (Cd, Cu, Zn, Sb) are more closely linked to diffuse urban sources such as traffic. Pb and As occupy an intermediate position, reflecting multiple or mixed sources.

**Table 5:** Pearson correlation coefficients (r) among PTEs in surface soils from La Paz, Baja California Sur, Mexico

	As	Cd	Co	Cr	Cu	Ni	Pb	Sb	V	Zn
As	1.00**	0.53**	0.37	0.46*	0.16	− 0.02	0.23	0.47*	0.03	0.28
Cd		1.00**	0.11	0.53**	0.71**	0.20	0.45*	0.14	0.21	0.71**
Co			1.00**	0.29	0.17	0.09	− 0.02	0.20	0.42*	0.16
Cr				1.00**	0.37	0.59**	− 0.13	0.22	0.67**	0.28
Cu					1.00**	0.26	0.46*	0.06	0.36	0.64**
Ni						1.00**	− 0.13	0.16	0.87**	0.39
Pb							1.00**	0.04	− 0.13	0.63**
Sb								1.00**	0.09	0.09
V									1.00**	0.42*
Zn										1.00**

^**^: Correlation is significant at the 0.01 level (two-tailed)

^*^: Correlation is significant at the 0.05 level (two-tailed)

**Fig. 6:**
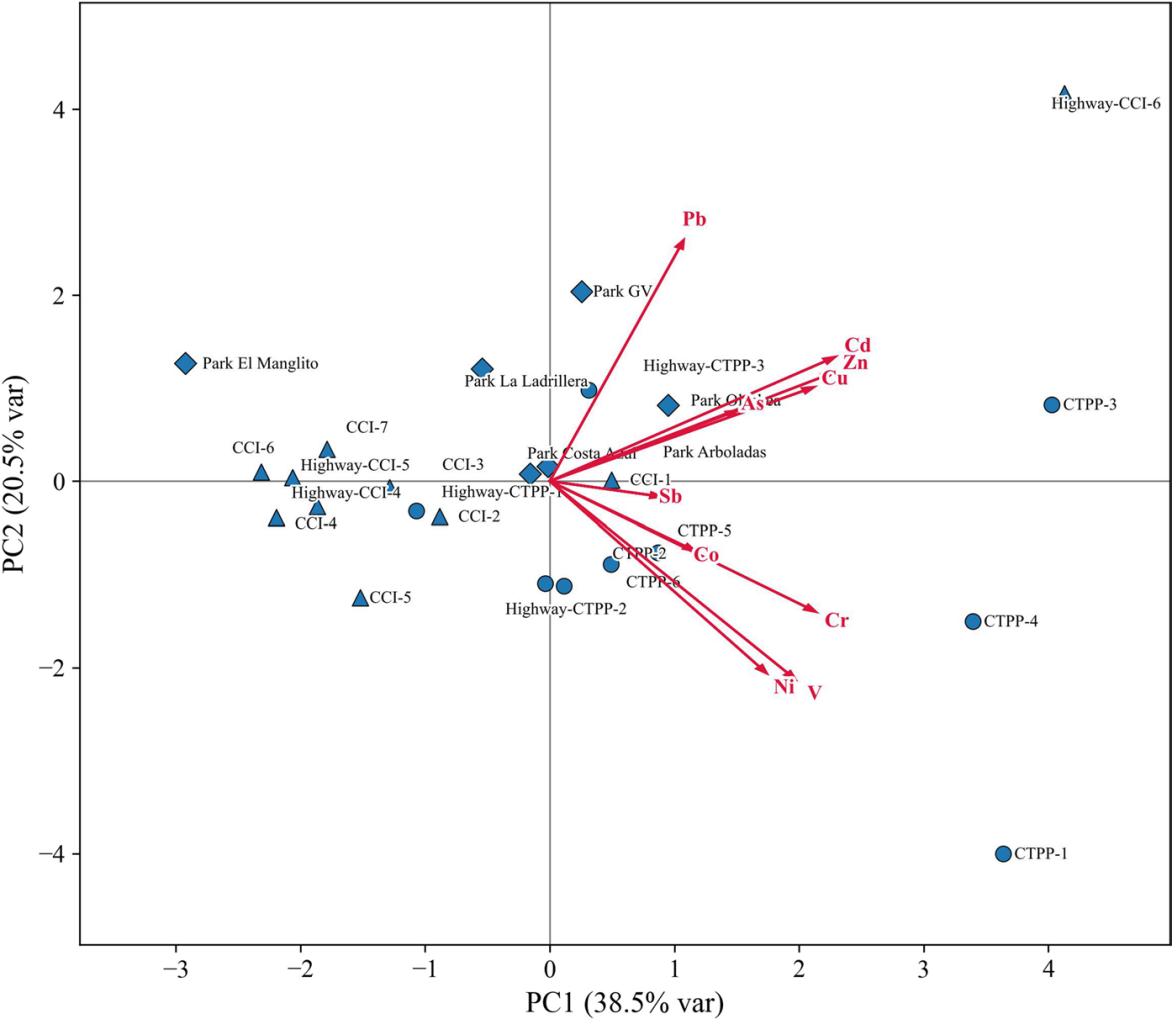
Principal component analysis (PCA) biplot showing the relationships among PTEs and sampling sites in La Paz soils. Red vectors represent the loading of each element in the first two principal components (PC1 and PC2), explaining 59% of total variance

PCA results (Fig. 6 and Table S7) further refined these associations, identifying four main groupings based on similar loading patterns. As, Cu, Cd, and Zn formed the first group, all with high positive loadings on PC1 (0.53–0.81) and moderate loadings on PC2, suggesting similar spatial distributions and potentially shared sources. Their enrichment in residential and traffic-affected areas points to diffuse urban emissions, including vehicular wear products, with possible localized industrial inputs. Ni and V constituted the second group, both with moderately high positive loadings on PC1 (0.62–0.70) and high negative loadings on PC2 (− 0.63 and − 0.65). Their strong correlation and spatial concentration near the CTPP and CCI facilities are consistent with emissions from oil combustion in power generation. A third group comprised Cr, Co, and Sb, with positive loadings on PC1 (0.32–0.76) and negative loadings on PC2 (− 0.05 to − 0.43). Although correlations within this group were weaker than in the first two, their proximity in the PCA plot suggests partially overlapping sources or similar geochemical behavior. The widespread occurrence of Sb, a recognized traffic tracer, combined with the distribution of Cr and Co, points to contributions from both vehicular emissions and minor industrial sources. Pb displayed a distinct behavior, with a relatively low loading on PC1 (0.38) but the highest loading on PC2 (0.79), clearly separating it from the other groups in the PCA space. Its distribution, with moderate enrichment in both traffic-affected and industrial-adjacent areas, indicates multiple inputs, including historical deposition and ongoing emissions.

Overall, the integration of spatial and statistical evidence identifies four coherent associations: (1) As–Cu–Cd–Zn linked to diffuse urban and traffic sources, (2) Ni–V associated with industrial oil combustion, (3) Cr–Co–Sb reflecting mixed traffic and industrial influences, and (4) Pb as a distinct marker of multiple and possibly historical sources. These findings highlight the complementary value of spatial mapping and multivariate statistics for disentangling the complex mixture of point-source and diffuse contamination in La Paz soils.

### Evaluation of human health risks

Non-carcinogenic hazard index (HI) values for individual PTEs (Table 6) showed that, for adults, all values remained well below the threshold of 1 across the four sites. The highest contributions were observed at CTPP for As (6.32E-02), Co (6.17E-02), and V (4.34E-02), followed by Pb (2.51E-02). At CCI, As (5.33E-02), Co (5.17E-02), Zn (4.78E-02), and Pb (3.12E-02) were the dominant contributors. The Highway site was characterized by elevated As (6.27E-02), Co (5.45 E-02), Pb (4.81 E-02), and V (2.39 E-02), while Park soils presented As (6.12 E-02), Co (4.98 E-02), Pb (4.69 E-02), and V (1.65 E-02) as the main contributors. In children, individual HI values were consistently higher than in adults, although no single metal exceeded the threshold of 1. The greatest contributors were found at CTPP (As = 5.88E-01, Co = 5.73E-01, V = 3.84E-01, Cr = 2.91E-01, Pb = 2.34E-01), followed by CCI (As = 4.96E-01, Co = 4.80E-01, Pb = 2.91E-01, V = 2.10E-01), Highway (As = 5.84E-01, Co = 5.06E-01, Pb = 4.49E-01, V = 2.11E-01), and Park (As = 5.70E-01, Co = 4.62E-01, Pb = 4.37E-01, V = 1.45E-01). Total carcinogenic risk (TCR) indicate that As and Cr were the primary contributors to in both adults and children. For adults, CTPP presented Cr = 2.00E-05 and As = 1.22E-05, followed by CCI (As = 1.03E-05, Cr = 9.58E-06), Highway (As = 1.21E-05, Cr = 1.25E-05), and Park (As = 1.18E-05, Cr = 1.05E-05). Minor contributions were recorded from Pb, Ni, and Cd. In children, maximum values were observed at CTPP (Cr = 3.73E-05, As = 2.27E-05), followed by Highway (Cr = 2.33E-05, As = 2.25E-05), Park (Cr = 1.96E-05, As = 2.20E-05), and CCI (Cr = 1.79E-05, As = 1.91E-05).

**Table 6:** Non-carcinogenic hazard index (HI) and total carcinogenic risks (TCR) for adults and children based on exposure to PTEs in surface soils from the four sampling sites in La Paz, Baja California Sur, Mexico

	As	Cd	Co	Cr	Cu	Ni	Pb	Sb	V	Zn
*Non-carcinogenic*										
*Adults*										
CTPP	6.32E-02	6.44E-04	6.17E-02	3.16E-02	1.07E-03	3.80E-03	2.51E-02	1.05E-02	4.34E-02	7.59E-04
CCI	5.33E-02	2.57E-04	5.17E-02	1.51E-02	5.11E-04	1.06E-03	3.12E-02	1.07E-02	2.37E-02	4.78E-02
Highway	6.27E-02	4.28E-04	5.45E-02	1.97E-04	8.81E-04	1.04E-03	4.81E-02	7.78E-03	2.39E-02	6.29E-04
Park	6.12E-02	4.26E-04	4.98E-02	1.66E-04	9.11E-04	8.14E-04	4.69E-02	1.35E-02	1.65E-02	5.83E-04
*Children*										
CTPP	5.88E-01	5.94E-03	5.73E-01	2.91E-01	9.93E-03	3.53E-02	2.34E-01	9.31E-02	3.84E-01	7.07E-03
CCI	4.96E-01	2.37E-03	4.80E-01	1.39E-01	4.76E-03	9.82E-03	2.91E-01	9.47E-02	2.10E-01	4.46E-03
Highway	5.84E-01	3.94E-03	5.06E-01	1.82E-01	8.21E-03	9.63E-03	4.49E-01	6.89E-02	2.11E-01	5.86E-03
Park	5.70E-01	3.92E-03	4.62E-01	1.53E-01	8.49E-03	7.57E-03	4.37E-01	1.20E-01	1.45E-01	5.43E-03
*Carcinogenic*										
*Adults*										
CTPP	1.22E-05	2.51E-10	–	2.00E-05	–	3.97E-09	1.27E-07	–	–	–
CCI	1.03E-05	1.00E-10	–	9.58E-06	–	1.10E-09	1.59E-07	–	–	–
Highway	1.21E-05	1.67E-10	–	1.25E-05	–	1.08E-09	2.45E-07	–	–	–
Park	1.18E-05	1.66E-10	–	1.05E-05	–	8.49E-10	2.38E-07	–	–	–
*Children*										
CTPP	2.27E-05	8.91E-11	–	3.73E-05	–	1.41E-09	2.38E-07	–	–	–
CCI	1.91E-05	3.56E-11	–	1.79E-05	–	3.91E-10	2.96E-07	–	–	–
Highway	2.25E-05	5.92E-11	–	2.33E-05	–	3.83E-10	4.57E-07	–	–	–
Park	2.20E-05	5.88E-11	–	1.96E-05	–	3.01E-10	4.45E-07	–	–	–

The cumulative HI values for adults were 0.24 (CTPP), 0.19 (CCI), 0.22 (Highway), and 0.21 (Park), all well below 1. In contrast, children exhibited cumulative HI values above the threshold in all sites: 2.22 (CTPP), 1.73 (CCI), 2.03 (Highway), and 1.91 (Park). Cumulative TCR values for adults ranged from 2.00E-05 (CCI) to 3.23E-05 (CTPP), whereas in children they ranged from 3.73E-05 (CCI) to 6.02E-05 (CTPP). All cumulative TCR values fell within the generally acceptable risk range (10^−6^ to 10^−4^).

The results indicate no non-carcinogenic risk for adults, whereas cumulative HI values above 1 in children across all sites point to potential non-carcinogenic concern, particularly at CTPP, where contributions from As, Co, V, Cr, and Pb were the highest. Although carcinogenic risks for both adults and children remain within the acceptable range, values at CTPP approach the upper bound of the tolerance range, primarily due to Cr and As. These findings suggest that children are more vulnerable to soil-borne PTE exposure in the study area. The elevated risk near CTPP is consistent with localized industrial emissions, whereas Highway and Park sites also reflect diffuse contributions from vehicular traffic and urban deposition. This evidence underscores the need for continued monitoring, targeted mitigation strategies near industrial sources, and preventive measures to reduce direct soil contact, particularly for children in high-exposure areas.

## Limitations

This study has certain limitations that should be considered. The sampling campaign represents a single temporal snapshot, which restricts the ability to capture seasonal variability in atmospheric deposition, soil resuspension, or surface disturbance. The use of total digestion provides the complete elemental burden but does not differentiate between bioavailable, mobile, or speciated fractions, factors that can influence ecological and health risk assessments. In addition, although the combination of spatial analyses and multivariate statistics strengthened source identification, the absence of direct atmospheric emission or deposition measurements introduces uncertainty in distinguishing overlapping industrial and urban contributions. Future research incorporating temporal monitoring, chemical fractionation, and atmospheric dispersion modeling would help refine source attribution and improve the characterization of exposure pathways.

## Conclusions

This study evaluated the distribution, sources, and potential risks of PTEs in surface soils of La Paz, Baja California Sur. The results show that industrial emissions, particularly from heavy fuel oil combustion at the CTPP and CCI power plants, together with diffuse urban inputs from traffic, shape the spatial patterns of metals in the region. Although most concentrations comply with national and international soil quality guidelines, V in park soils and both V and Ni near the power plants displayed notable enrichment, indicating a measurable influence from industrial activities. Traffic-related tracers such as Sb, Zn, and Cu were elevated along major roads and in urban parks, while mixed-source elements (e.g., Cr, Pb, As) reflected the combined effect of industrial operations, local transit, and land-use patterns. Multivariate and magnetic analyses supported this source differentiation, reinforcing the role of oil combustion and vehicular wear as the dominant contributors. From a human health perspective, non-carcinogenic risks were negligible for adults but exceeded the safety threshold for children across all land uses, highlighting the vulnerability of younger populations. Cancer risks remained within the acceptable range yet approached the upper limit near the CTPP.

Overall, the findings indicate that soil quality in La Paz is generally within regulatory limits but exhibits clear signatures of industrial and urban emissions. Continued monitoring, particularly of V, Ni, and As, and targeted mitigation strategies are recommended to minimize long-term exposure risks and to support evidence-based environmental management.

## Supplementary Information

Below is the link to the electronic supplementary material.

Supplementary file1 (DOCX 839 kb)

## Data Availability

No datasets were generated or analysed during the current study.
