# Supplementary material for: Spatial distribution, contamination levels, and health risk of potentially toxic elements in surface soils of an industrial—urban area in northwestern Mexico: a case study from La Paz, Baja California Sur
Source: Environ Geochem Health. 2025 Dec 9;48(1):36. doi: 10.1007/s10653-025-02918-7 (PMC12689732; doi:10.1007/s10653-025-02918-7)
Supplement: Supplementary file 1 — Supplementary file1 (DOCX 839 kb) [file 10653_2025_2918_MOESM1_ESM.docx]

**Table S1:** Quality control of results obtained with ICP-MS using SRM-1648.

|  | **SRM-1648** | **ICP-MS** |
| --- | --- | --- |
| **Potentially Toxic elements**  **(mg kg^-1^)** |  |  |
| As | 115.5 | 127.5 |
| Cd | 73.7 | 71.1 |
| Co | 17.93 | 19.57 |
| Cr | 402 | 446.31 |
| Cu | 610 | 620.1 |
| Ni | 81.1 | 81.78 |
| Pb | 6550 | 6541.9 |
| Sb | 45.4 | 46.8 |
| V | 127 | 126.32 |
| Zn | 4800 | 4548.8 |

**Table S2:** Parameters and values for human health risk calculation (USEPA, 2002; Cakmak et al., 2020).

|  |  |  | Value | |
| --- | --- | --- | --- | --- |
| Factor | Unit | Definition | Children | Adults |
| ADD | mg kg^-1^ day^-1^ | Average daily dose through  ingestion, inhalation, and dermal contact | - | - |
| C | mg kg^-1^ | Concentration of metal(oid) | - | - |
| IngR | mg day^-1^ | Ingestion rate | 200 | 100 |
| InhR | m^3^ day^-1^ | Inhalation rate | 7.6 | 20 |
| EF | days year^-1^ | Exposure frequency | 350 | 350 |
| ED | years | Exposure duration | 6 | 24 |
| BW | kg | Body weight | 10 | 55 |
| AT_nc_ | days | Average time (for non-carcinogens) | 365×ED | 365×ED |
| AT_c_ | days | Average time (for carcinogens) | 365×70 | 365×70 |
| CF | kg mg^-1^ | Conversion factor | 1×10^-6^ | 1×10^-6^ |
| SA | cm^2^ | Surface skin area available  for exposure | 2800 | 5700 |
| AF | mg cm^-2^ day^-1^ | Skin adherence factor | 0.2 | 0.07 |
| ABF | - | Dermal adsorption factor | 0.001 | 0.001 |
| PEF | m^3^ kg^-1^ | Particle emission factor | 1.36×10^9^ | 1.36×10^9^ |

**Table S3:** Reference dose (RfD, mg kg^-1^ day^-1^) values for non-carcinogenic risk and slope factor (SF, kg mg^-1^ day^-1^) values for carcinogenic risk (USEPA, 2002; USEPA, 2010; Cakmak et al., 2020; Dat et al., 2021; Ferreira-Baptista and De Miguel, 2005; Khan et al., 2023).

| **Metal(loid)** |  | **RfD** |  |  |  | **SF** |  |
| --- | --- | --- | --- | --- | --- | --- | --- |
|  | Ingestion | Inhalation | Dermal contact |  | Ingestion | Inhalation | Dermal contact |
| As | 3.0E-04 | 5.0E-04 | 1.2E-04 |  | 1.5E+00 | 1.5E+01 | 3.7E+00 |
| Cd | 1.0E-03 | 1.0E-05 | 2.5E-05 |  | - | 6.3E+00 | - |
| Co | 3.0E-04 | 6.0E-05 | 9.0E-05 |  | - | - | - |
| Cr | 3.0E-03 | 2.7E-05 | 1.9E-02 |  | 5.0E-01 | 4.1E+01 | - |
| Cu | 4.0E-02 | 1.2E-02 | 4.0E-02 |  | - | - | - |
| Ni | 2.0E-02 | 5.4E-03 | 2.0E-02 |  | - | 8.4E-01 | - |
| Pb | 1.4E-03 | 3.5E-03 | 1.4E-03 |  | 8.5E-03 | 4.2E-02 | - |
| Sb | 4.0E-04 | 2.0E-05 | 8.0E-05 |  | - | - | - |
| V | 9.0E-03 | 2.0E-04 | 9.0E-03 |  | - | - | - |
| Zn | 3.0E-01 | 3.5E+01 | 3.0E-01 |  | - | - | - |

**Table S4:** Summary statistics (mean, minimum, maximum, median, and standard deviation) of PTEs concentrations (mg kg^−^¹) in soils collected from four distinct site categories: CTPP, CCI, Highway, and Park areas in La Paz, Baja California Sur, Mexico.

| **Trace Metal** | **Group** | **Mean** | **Min** | **Max** | **Median** | **SD** |
| --- | --- | --- | --- | --- | --- | --- |
| **As** | CTPP | 13.71 | 1.48 | 23.84 | 13.96 | 7.18 |
|  | CCI | 11.57 | 3.53 | 18.58 | 12.28 | 4.45 |
|  | Highway | 13.60 | 9.73 | 18.62 | 12.75 | 4.00 |
|  | Park | 13.28 | 6.14 | 16.28 | 14.37 | 3.60 |
| **Cd** | CTPP | 0.46 | 0.25 | 0.88 | 0.42 | 0.24 |
|  | CCI | 0.18 | 0.11 | 0.26 | 0.18 | 0.06 |
|  | Highway | 0.31 | 0.15 | 0.72 | 0.21 | 0.22 |
|  | Park | 0.31 | 0.21 | 0.39 | 0.33 | 0.07 |
| **Co** | CTPP | 13.42 | 8.96 | 22.37 | 12.95 | 4.83 |
|  | CCI | 11.24 | 7.95 | 15.20 | 10.98 | 2.22 |
|  | Highway | 11.85 | 7.69 | 19.71 | 11.10 | 4.36 |
|  | Park | 10.82 | 5.53 | 14.25 | 11.04 | 3.45 |
| **Cr** | CTPP | 68.10 | 46.11 | 92.48 | 67.63 | 19.48 |
|  | CCI | 32.62 | 23.94 | 44.51 | 32.38 | 6.61 |
|  | Highway | 42.46 | 31.44 | 50.87 | 43.27 | 8.33 |
|  | Park | 35.74 | 15.97 | 46.64 | 38.00 | 12.05 |
| **Cu** | CTPP | 30.98 | 20.71 | 45.21 | 28.35 | 10.42 |
|  | CCI | 14.85 | 9.50 | 22.70 | 14.60 | 4.05 |
|  | Highway | 25.60 | 13.51 | 46.00 | 17.94 | 15.09 |
|  | Park | 26.49 | 17.89 | 35.59 | 25.38 | 5.94 |
| **Ni** | CTPP | 54.73 | 13.30 | 129.70 | 37.87 | 47.66 |
|  | CCI | 15.20 | 9.74 | 27.32 | 13.37 | 5.94 |
|  | Highway | 14.90 | 12.83 | 17.85 | 14.22 | 1.91 |
|  | Park | 11.71 | 5.51 | 17.15 | 12.09 | 3.72 |
| **Pb** | CTPP | 25.51 | 14.05 | 31.45 | 26.86 | 6.15 |
|  | CCI | 31.76 | 23.13 | 42.86 | 30.27 | 6.30 |
|  | Highway | 49.00 | 21.92 | 142.70 | 27.26 | 47.07 |
|  | Park | 47.73 | 36.21 | 65.94 | 46.00 | 10.96 |
| **Sb** | CTPP | 2.55 | 0.69 | 3.75 | 2.85 | 1.16 |
|  | CCI | 2.60 | 1.38 | 5.90 | 2.09 | 1.61 |
|  | Highway | 1.89 | 0.96 | 2.61 | 2.09 | 0.65 |
|  | Park | 3.29 | 1.30 | 4.98 | 3.51 | 1.28 |
| **V** | CTPP | 182.55 | 94.50 | 302.80 | 170.40 | 71.23 |
|  | CCI | 99.90 | 46.92 | 156.20 | 99.08 | 38.50 |
|  | Highway | 100.38 | 63.15 | 137.38 | 96.56 | 26.37 |
|  | Park | 69.20 | 30.92 | 97.79 | 75.33 | 25.17 |
| **Zn** | CTPP | 165.55 | 62.53 | 274.57 | 172.36 | 78.12 |
|  | CCI | 104.36 | 71.47 | 145.63 | 108.57 | 24.22 |
|  | Highway | 137.10 | 87.11 | 325.95 | 102.96 | 92.98 |
|  | Park | 127.15 | 61.55 | 218.27 | 121.12 | 52.92 |

CTPP: Punta Prieta Thermoelectric Power Plant; CCI: Internal Combustion Power Plant); SD: Standard Deviation

**Table S5:** Preparation parameters for magnetic susceptibility measurements of surface soil samples collected from four site categories in La Paz: CTPP, CCI, Highway corridors, and urban parks.

| **Sample** | **Cube volume**  **×10^-6^(m^3^)** | | **Mass**  **(kg)** | **ρ ×10^3^**  **(kg m^-3^)** |
| --- | --- | --- | --- | --- |
| CTPP-1 | | 8.00 | 0.01127 | 1.41 |
| CTPP-2 | | 8.00 | 0.01199 | 1.50 |
| CTPP-3 | | 8.00 | 0.01241 | 1.55 |
| CTPP-4 | | 8.00 | 0.01091 | 1.36 |
| CTPP-5 | | 8.00 | 0.01259 | 1.57 |
| CTPP-6 | | 8.00 | 0.0134 | 1.68 |
| CCI-1 | | 8.00 | 0.01235 | 1.54 |
| CCI-2 | | 8.00 | 0.01331 | 1.66 |
| CCI-3 | | 8.00 | 0.011 | 1.38 |
| CCI-4 | | 8.00 | 0.01113 | 1.39 |
| CCI-5 | | 8.00 | 0.01227 | 1.53 |
| CCI-6 | | 8.00 | 0.01287 | 1.61 |
| CCI-7 | | 8.00 | 0.01311 | 1.64 |
| Highway-CTPP-1 | | 8.00 | 0.01154 | 1.44 |
| Highway-CTPP-2 | | 8.00 | 0.01212 | 1.52 |
| Highway-CTPP-3 | | 8.00 | 0.01186 | 1.48 |
| Highway-CCI-1 | | 8.00 | 0.01384 | 1.73 |
| Highway-CCI-2 | | 8.00 | 0.01311 | 1.64 |
| Highway-CCI-3 | | 8.00 | 0.01384 | 1.73 |
| Park Arboledas | | 8.00 | 0.01257 | 1.57 |
| Park El Manglito | | 8.00 | 0.01236 | 1.55 |
| Park Olachea | | 8.00 | 0.01425 | 1.78 |
| Park Ladrillera | | 8.00 | 0.01284 | 1.61 |
| Park Costa Azul | | 8.00 | 0.01294 | 1.62 |
| Park GV | | 8.00 | 0.0143 | 1.79 |
| Background | | 8.00 | 0.01246 | 1.56 |

**Table S6:** Descriptive statistics (mean, minimum, maximum, median, and standard deviation) of contamination indices for PTEs in urban soils from La Paz, Mexico.

|  |  | **As** | **Cd** | **Co** | **Cr** | **Cu** | **Ni** | **Pb** | **Sb** | **V** | **Zn** |
| --- | --- | --- | --- | --- | --- | --- | --- | --- | --- | --- | --- |
| **CF** | Mean | 1.31 | 3.09 | 1.36 | 1.68 | 1.67 | 2.22 | 1.44 | 3.40 | 1.41 | 1.60 |
|  | Minimum | 0.15 | 1.10 | 0.64 | 0.61 | 0.66 | 0.51 | 0.53 | 0.91 | 0.39 | 0.74 |
|  | Maximum | 2.40 | 8.80 | 2.59 | 3.51 | 3.18 | 12.11 | 5.36 | 7.76 | 3.80 | 3.93 |
|  | Median | 1.37 | 2.60 | 1.30 | 1.65 | 1.47 | 1.31 | 1.14 | 3.25 | 1.23 | 1.32 |
|  | SD | 0.48 | 1.84 | 0.43 | 0.69 | 0.76 | 2.65 | 0.93 | 1.67 | 0.74 | 0.79 |
| **Igeo** | Mean | -0.36 | 0.85 | -0.20 | 0.05 | 0.02 | 0.10 | -0.23 | 1 | -0.26 | -0.05 |
|  | Minimum | -3.33 | -0.45 | -1.23 | -1.31 | -1.19 | -1.54 | -1.51 | -0.72 | -1.95 | -1.01 |
|  | Maximum | 0.68 | 2.55 | 0.79 | 1.23 | 1.09 | 3.01 | 1.84 | 2.37 | 1.34 | 1.39 |
|  | Median | -0.14 | 0.79 | -0.20 | 0.14 | -0.03 | -0.19 | -0.40 | 1.12 | -0.29 | -0.18 |
|  | SD | 0.82 | 0.74 | 0.43 | 0.57 | 0.64 | 0.99 | 0.64 | 0.75 | 0.73 | 0.62 |
| **Er** | Mean | 13.09 | 92.76 | 6.82 | 3.36 | 8.33 | 11.10 | 7.18 | 33.98 | 2.82 | 1.60 |
|  | Minimum | 1.50 | 33 | 3.20 | 1.22 | 3.30 | 2.55 | 2.65 | 9.10 | 0.78 | 0.74 |
|  | Maximum | 24 | 264 | 12.95 | 7.02 | 15.90 | 60.55 | 26.80 | 77.60 | 7.60 | 3.93 |
|  | Median | 13.70 | 78 | 6.50 | 3.30 | 7.35 | 6.55 | 5.70 | 32.50 | 2.46 | 1.32 |
|  | SD | 4.78 | 55.09 | 2.13 | 1.39 | 3.80 | 13.23 | 4.63 | 16.67 | 1.48 | 0.79 |

**Table S7:** Principal component loadings for PTEs in surface soils of La Paz, Baja California Sur, Mexico. Underlined values indicate strong loadings (|loading| ≥ 0.70) for each principal component (PC), used to infer potential common sources of contamination.

|  | **PC1** | **PC2** | **PC3** |
| --- | --- | --- | --- |
| **As** | 0.53372349 | 0.23231598 | -0.7317465 |
| **Cd** | 0.81108772 | 0.4087682 | 0.00542285 |
| **Co** | 0.41225635 | -0.2280034 | -0.4416459 |
| **Cr** | 0.75591879 | -0.4283064 | -0.1561034 |
| **Cu** | 0.75070844 | 0.30946001 | 0.29974729 |
| **Ni** | 0.61671144 | -0.6307705 | 0.3214498 |
| **Pb** | 0.38072233 | 0.79353688 | 0.16557839 |
| **Sb** | 0.31825346 | -0.0511386 | -0.6688979 |
| **V** | 0.69783685 | -0.6502525 | 0.24820709 |
| **Zn** | 0.8051113 | 0.35627178 | 0.29758836 |

**Figure S1:** Location of the main industrial facilities in the study region, including the Punta Prieta Thermoelectric Power Plant (CTPP) and the Internal Combustion Power Plant (CCI). The insets provide detailed satellite views of each industrial complex and the corresponding sampling sites.

**
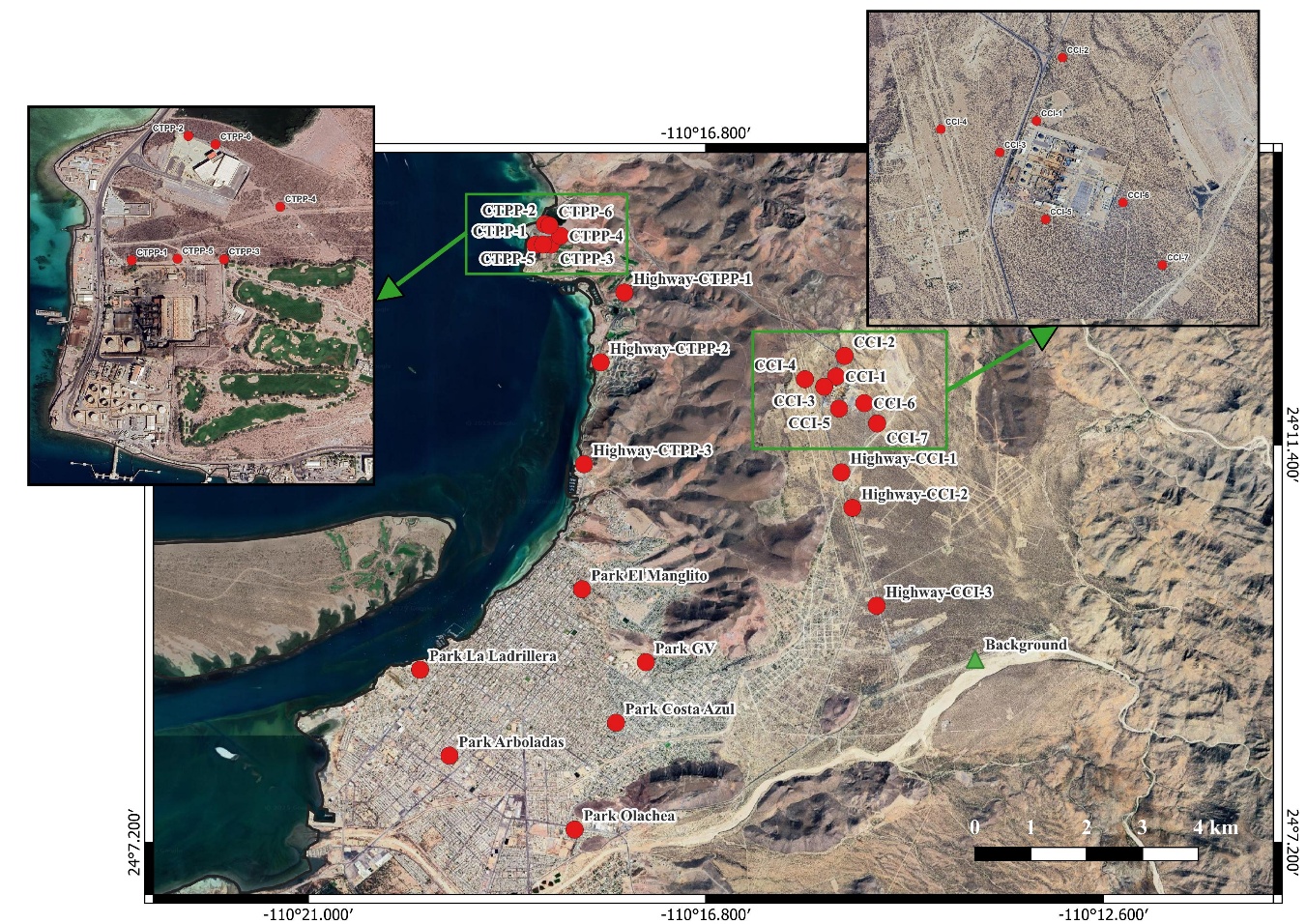
**

**Figure S2:** Pearson correlation matrix of PTEs in surface soils from La Paz, Baja California Sur, Mexico. Correlation coefficients are shown in a color-coded scale, with significant positive correlations (p < 0.05) indicated by asterisks. Darker shades correspond to stronger associations.


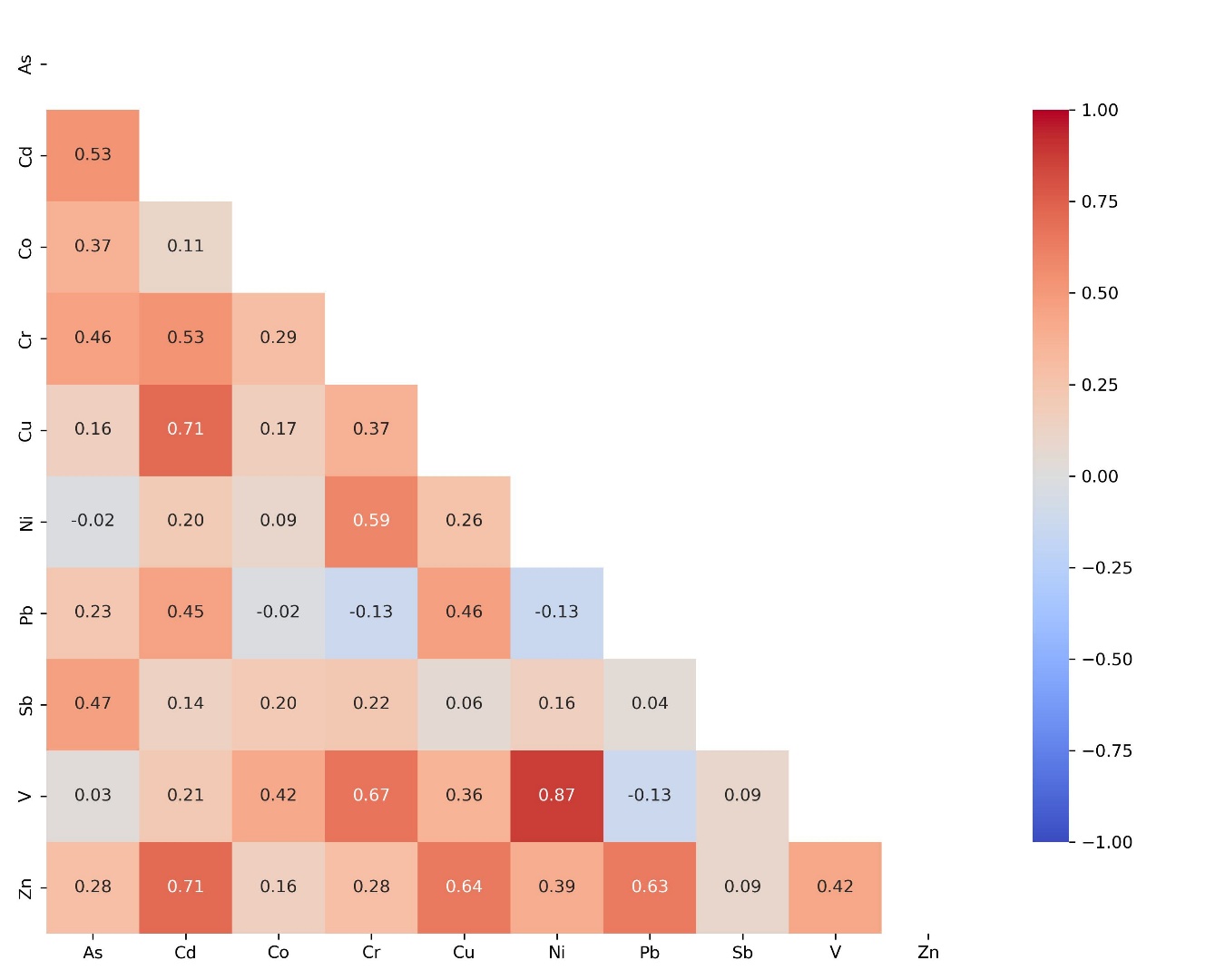


**References**

Čakmak, D., Perović, V., Kresović, M. et al. (2020). Sources and a Health Risk Assessment of Potentially Toxic Elements in Dust at Children’s Playgrounds with Artificial Surfaces: A Case Study in Belgrade. Arch Environ Contam Toxicol 78, 190–205. <https://doi.org/10.1007/s00244-019-00702-0>

Dat, N.D., Nguyen, VT., Vo, TDH. et al. (2021). Contamination, source attribution, and potential health risks of heavy metals in street dust of a metropolitan area in Southern Vietnam. Environ Sci Pollut Res 28, 50405–50419.

[https://doi.org/10.1007/s11356- 021-14246-1](https://doi.org/10.1007/s11356-%09021-14246-1)

Ferreira-Baptista, L., and De Miguel, E. (2005). Geochemistry and risk Assessment od street dust in Luanda, Angola: A tropical urban environment. Atmospheric Environment, 39, 4501-4512. <https://doi.org/10.1016/j.atmosenv.2005.03.026>

Khan, M., Setu, S., Sultana, N. et al. (2023). Street dust in the largest urban agglomeration: pollution characteristics, source apportionment and health risk assessment of potentially toxic trace elements. Stoch Environ Res Risk Assess 37, 3305–3324. <https://doi.org/10.1007/s00477-023-02432-1>

USEPA (2002) Supplemental Guidance for Developing Soil Screening Levels for Superfund Sites. The U.S. Environmental Protection Agency, Office of Emergency and Remedial Response, Washington, DC.

USEPA (2010) Integrated Risk Information System (IRIS); United States Environmental Protection Agency. USEPA (United States Environmental Protection Agency)., Washington.
